# Cleavage of DNA and RNA by PLD3 and PLD4 limits autoinflammatory triggering by multiple sensors

**DOI:** 10.1038/s41467-021-26150-w

**Published:** 2021-10-07

**Authors:** Amanda L. Gavin, Deli Huang, Tanya R. Blane, Therese C. Thinnes, Yusuke Murakami, Ryutaro Fukui, Kensuke Miyake, David Nemazee

**Affiliations:** 1 The Department of Immunology and Microbiology, The Scripps Research Institute, La Jolla, CA 92037 USA; 2 Laboratory of Pharmacotherapy, Faculty of Pharmacy, Musashino University, Tokyo, Japan; 3 Division of Innate Immunity, Department of Microbiology and Immunology, The Institute of Medical Science, The University of Tokyo, Tokyo, 108-8639 Japan

**Keywords:** Interferons, Acute inflammation, Toll-like receptors

## Abstract

Phospholipase D3 (PLD3) and PLD4 polymorphisms have been associated with several important inflammatory diseases. Here, we show that PLD3 and PLD4 digest ssRNA in addition to ssDNA as reported previously. Moreover, *Pld3*^−/−^*Pld4*^−/−^ mice accumulate small ssRNAs and develop spontaneous fatal hemophagocytic lymphohistiocytosis (HLH) characterized by inflammatory liver damage and overproduction of Interferon (IFN)-γ. Pathology is rescued in *Unc93b1*^3d/3d^*Pld3*^−/−^*Pld4*^−/−^ mice, which lack all endosomal TLR signaling; genetic codeficiency or antibody blockade of TLR9 or TLR7 ameliorates disease less effectively, suggesting that both RNA and DNA sensing by TLRs contributes to inflammation. IFN-γ made a minor contribution to pathology. Elevated type I IFN and some other remaining perturbations in *Unc93b1*^3d/3d^*Pld3*^−/−^*Pld4*^−/−^ mice requires STING (*Tmem173*). Our results show that PLD3 and PLD4 regulate both endosomal TLR and cytoplasmic/STING nucleic acid sensing pathways and have implications for the treatment of nucleic acid-driven inflammatory disease.

## Abstract (web summary)

Loss of function polymorphisms of phospholipase D3 and D4 are associated with inflammatory diseases and their function is unclear. Here the authors show that PLD3/4 function as RNAses and deletion of these proteins in mice leads to accumulation of ssRNA which exacerbates inflammation through TLR signalling.

## Introduction

Nucleic acid sensing is important in host responses to infection, tissue damage, and tumors^1,2^, promoting activation of a variety of cell types and the production of IFNs and inflammatory cytokines. Some nucleic acid sensors, such as RIG-I, MDA5, cGAS, and AIM2, reside in the cytoplasm and signal through pathways involving MAVS, STING, or inflammasomes^1,3^. Nucleic acid-sensing TLRs, including TLRs 3, 7, 8, 9, and 13, recognize fragments of DNA and RNA in endosomes and lysosomes, the processing of which is still incompletely understood^4–10^. TLR3 recognizes double-stranded (ds) RNA, TLR7 and 8 recognize ssRNA, TLR9 senses ssDNA carrying unmethylated CpG motifs, and TLR13 recognizes a sequence from bacterial 23S ribosomal RNA. TLRs 7, 8, 9, and 13 signal through the MyD88 pathway, leading to activation of NF-κB and IRF pathways, whereas TLR3 triggers those pathways through the adapter TRIF^11^.

Defects in host nucleases can lead to autoinflammatory or autoimmune syndromes^12,13^ but none so far mimic the symptoms of hemophagocytic lymphohistiocytosis (HLH). The familial form (FLH) is often associated with defects in cytotoxic killing by CD8^+^ T cells or NK cells^14^. Secondary or sporadic HLH, also called “macrophage activation syndrome” (MAS), is associated with infection, neoplasia, autoimmunity, and autoinflammatory disorders^15,16^. Characteristic features include hemophagocytosis, elevated cytokine levels, fever, hepatosplenomegaly, liver dysfunction, anemia, cytopenia, low NK cell activity, thrombocytopenia, weight loss, and elevated levels of ferritin and other acute-phase proteins^15,16^. HLH is often caused by infection-triggered hyperactivation, which produces a toxic “cytokine storm”. HLH can also be triggered by SARS-CoV-2^17^. Overall, HLH and MAS describe a range of hyperinflammatory syndromes with a common terminal pathway but diverse pathogenic origins^16,18^.

PLD3 and PLD4 are phospholipase D family proteins with associations to human diseases. Genome-wide association studies have linked *PLD4* polymorphisms to systemic sclerosis, systemic lupus erythematosus and rheumatoid arthritis^19–21^. PLD3 is structurally similar to PLD4. Missense mutations of *PLD3* or its under-expression have been implicated in late-onset Alzheimer’s disease and in spinocerebellar ataxia^22–25^. Although PLD3 has been suggested to be an active phospholipase^23,26,27^, the evidence is indirect and controversial^28,29^. We previously discovered that PLD3 and PLD4 are endolysosomal ssDNA exonucleases that limit TLR9 responses^30^ and that *Pld3*^−/−^*Pld4*^−/−^ double-deficient mice developed a spontaneous, fatal HLH-like disease^16,30^.

Here we study the function of PLD3 and PLD4 in degrading ssRNA and suppressing nucleic acid sensing. We test whether endosomal nucleic acid sensing in general or TLR9, in particular, is necessary for disease, and we assess the contribution of IFN-γ to pathology. We find that PLD3 and PLD4 have ssRNA exonuclease activity, and that the disease of mice deficient in both PLD3 and PLD4 has contributions by TLR7 and possibly other endosomal TLRs and a sensor that triggers the STING pathway.

## Results

### The contribution of IFN-γ to disease in *Pld3*^−/−^*Pld4*^−/−^ mice

*Pld3*^−/−^*Pld4*^−/−^ mice on a C57BL/6 background die at 2–3 weeks of age of HLH^30^ (Supplementary Fig. 1). Because IFN-γ is a signature cytokine of HLH^15,31^, we first tested the extent to which *Ifng*^*−/−*^*Pld3*^−/−^*Pld4*^−/−^ mice were protected from the disease compared to *Pld3*^−/−^*Pld4*^−/−^ mice. Although *Ifng*^*−/−*^*Pld3*^−/−^*Pld4*^−/−^ mice survived somewhat longer than *Pld3*^−/−^*Pld4*^−/−^ mice, they still died about 40 days after birth. At 2–3 weeks, *Ifng*^*−/−*^*Pld3*^−/−^*Pld4*^−/−^ mice showed substantial liver inflammation, anemia, thrombocytopenia, hemophagocytosis and myeloid cell infiltration within the liver, blood monocyte elevations, and strikingly reduced numbers of B cells, T cells and NK cells (Supplementary Fig. 1). This pathology was, however, distinguishable from the disease of *Pld3*^−/−^*Pld4*^−/−^ mice as microvesicular steatosis was less prominent and the livers contained large numbers of erythrocytes (Supplementary Fig. 2b). IFN-γ deficiency also did not alter the activation phenotype of CD4^+^ or CD8^+^ cells (Supplementary Fig. 1j-m), though NK-T cell numbers were significantly higher and had a KLRG1 + phenotype suggestive of activation (Supplementary Fig. 1 n,o). In summary, IFN-γ, while contributing somewhat to disease severity and phenotypes, was not required for the development of lethal HLH in *Pld3*^−/−^*Pld4*^−/−^ mice.

### Altered inflammatory phenotype of *Tlr9*^*−/−*^*Pld3*^−/−^*Pld4*^−/−^ mice

**Fig. 1:**
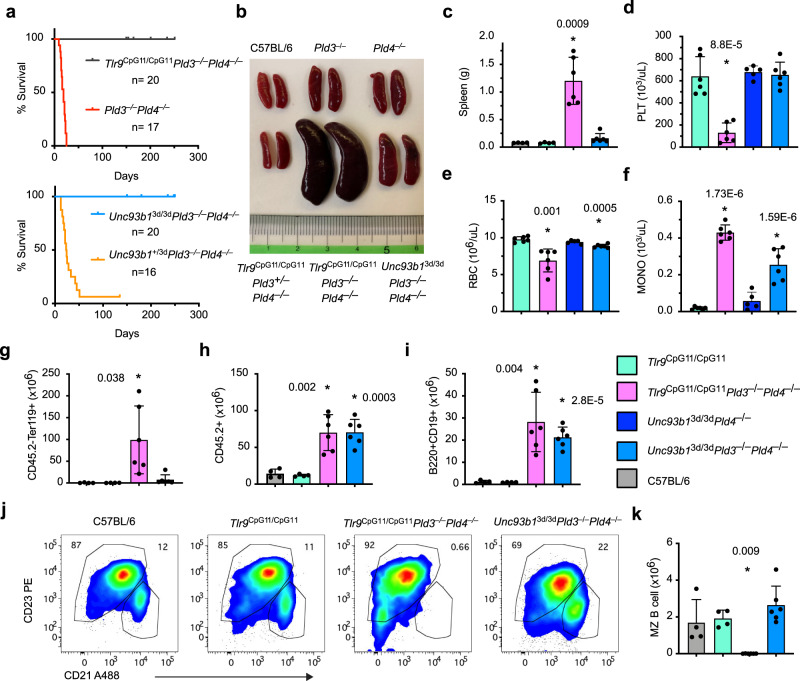
Immunological abnormalities and rescue of survival in *Tlr9*^CpG11/CpG11^*Pld3*^−/−^*Pld4*^−/−^ and *Unc93b1*^3d/3d^*Pld3*^−/−^*Pld4*^−/−^ mice. Mice of the indicated genotypes were analyzed for: **a** survival, **b**, **c** splenomegaly. Their blood was analyzed for numbers of **d** platelets, **e** erythrocytes, **f** monocytes. **g**–**k** Flow cytometry analysis for numbers of: **g** Splenic Ter119 + CD45− erythroblasts, **h** Total CD45.2+ leukocytes from lymph nodes, **i** B220^+^CD19^+^ B cells from lymph nodes, **j**, **k** splenic CD21^hi^CD23^lo^ marginal zone (MZ) B cells. Bar graphs show mean ± SD with each symbol representing an individual mouse. In **a**, survival of *Pld3*^−/−^*Pld4*^−/−^ mice (*n* = 17; median 19 d) and *Unc93b1*^+/3d^*Pld3*^−/−^*Pld4*^−/−^ mice (*n* = 16, median 23.5 d) was significantly shorter than survival of *Tlr9*^CpG11/CpG11^*Pld3*^−/−^*Pld4*^−/−^ (*n* = 20) and *Unc93b1*^3d/3d^*Pld3*^−/−^*Pld4*^−/−^ (*n* = 18) mice, among which there were no deaths at 250 d (Chi square 43.58 and 38.09, respectively, *p* < 0.0001, *p* < 0.0001 log rank test). In plots **c**, **g**, **h**, **i**, and **k**, unpaired, two-tailed *T*-tests were compared to C57BL/6 values, while for plots **d**–**f**, unpaired *T*-tests refer to comparisons with the *Tlr9*^CpG11/CpG11^ group. In **c**, **g**, **h**, **i**, **k** independent mouse numbers (*n*): C57BL/6 (4), *Tlr9*^CpG11/CpG11^ (4)*, Tlr9*^CpG11/CpG11^*Pld3*^*−/−*^*Pld4*^−/−^ (6)*, Unc93b*^−/−^*Pld3*^−/−^*Pld4*^−/−^ (6); in (**d**–**f**) independent mouse numbers (*n*) were *Tlr9*^CpG11/CpG11^ (6)*, Unc93b*^−/−^*Pld4*^−/−^ (5), *Tlr9*^CpG11/CpG11^*Pld3*^*−/−*^*Pld4*^−/−^ (6)*, Unc93b*^−/−^*Pld3*^−/−^*Pld4*^−/−^ (6). These experiments were performed at least twice with similar results.

As TLR9 appeared to drive all of the phenotypic features of *Pld4*^−/−^ mice^30^, we tested if it was also the sole sensor promoting inflammation in *Pld3*^−/−^*Pld4*^−/−^ mice. Here we tested initially the *Tlr9*^CpG11^ N-ethyl-N-nitrosourea-induced mutant allele, available on the C57BL/6 background (https://www.jax.org/strain/014534), which was used in the previous study. *Tlr9*^CpG11/CpG11^*Pld3*^−/−^*Pld4*^−/−^ mice rescued mouse survival (Fig. 1a), allowing a detailed comparison of young adult mice to other viable controls. Surprisingly, however, *Tlr9*^CpG11/CpG11^*Pld3*^−/−^*Pld4*^−/−^ mice were highly abnormal, as they had severe splenomegaly (Fig. 1b, c), with reduced erythrocytes and elevated monocyte proportions in the blood, and highly significant thrombocytopenia (Fig. 1d–f). In contrast to *Pld3*^−/−^*Pld4*^−/−^ liver tissue, analysis of *Tlr9*^CpG11/CpG11^*Pld3*^−/−^*Pld4*^−/−^ liver histopathology revealed a lack of microvesicular steatosis (Supplementary Fig. 2c). However, other histological abnormalities were still present, albeit less severe than in *Pld3*^−/−^*Pld4*^−/−^ mice, including infiltrates of CD68^+^ myeloid cells, hepatomegaly, multifocal medullary hematopoiesis, and hemophagocytosis (Supplementary Fig. 2c, d). In the spleen, Ter119^+^CD45^–^ erythroblasts were abundant, suggestive of significant extramedullary hematopoiesis (Fig. 1g). B cell numbers were restored (Fig. 1h, i), though they had altered phenotypes and the splenic CD19^+^B220^+^CD93^–^CD23^lo^CD21^+^ “marginal zone” B cell subset was absent (Fig. 1j, k). Whilst NK cell numbers remained reduced relative to WT controls, NK-T cell numbers were increased several-fold (Supplementary Fig. 3a, d, e). Most of the remaining T cells showed evidence of prior activation, including low CD62L expression and elevated surface expression of CD44 and MHCII (Supplementary Fig. 3b, c, f–h). Thus, in the absence of functional PLD3 and PLD4, signaling through TLR9 made a major contribution to disease severity. Consistent with these results, administration of TLR9 blocking mAb ameliorated disease even when given after severe wasting had occurred (Supplementary Fig. 4). Nevertheless, significant inflammation and hematopoietic dysregulation still occurred in *Tlr9*^CpG11/CpG11^*Pld3*^−/−^*Pld4*^−/−^ mice. As *Pld4*^−/−^*Tlr9*^CpG11/CpG11^ mice were healthy and similar phenotypically to *Tlr9*^CpG11/CpG11^ mice^30^, these data also underscore the observation that PLD3 plays an essential role in preventing autoinflammation in the absence of PLD4.

### Endosomal RNA sensing contributes to inflammation

To test whether RNA sensing in endolysosomes contributed to disease in the absence of PLD3 and PLD4, we introduced the *Unc93b1*^3d/3d^ mutation^32^, which prevents sensing through TLRs 3, 7, 9, 11, 12, and 13^5,32,33^. *Unc93b1*^3d/3d^*Pld3*^−/−^*Pld4*^−/−^ mice had a substantially normalized phenotype compared to *Pld3*^−/−^*Pld4*^−/−^ or *Tlr9*^CpG11/CpG11^*Pld3*^−/−^*Pld4*^−/−^ mice, as they survived for >8 months (Fig. 1a, lower panel) and lacked splenomegaly, anemia and thrombocytopenia (Fig. 1b–e). Marginal zone B cells, which were absent in *Tlr9*^CpG11/CpG11^*Pld3*^−/−^*Pld4*^−/−^ mice, were restored in *Unc93b1*^3d/3d^*Pld3*^−/−^*Pld4*^−/−^ mice (Fig. 1j, k). Hepatic histology, as judged by hematoxylin and eosin (H&E) stain, was essentially similar to WT (Supplementary Fig. 2c). Hemophagocytosis was absent in the liver and was also undetectable in the spleen using a flow cytometry assay^34^ (Supplementary Fig. 5a). However, *Unc93b1*^3d/3d^*Pld3*^−/−^*Pld4*^−/−^ mice showed several clear abnormalities in the numbers, frequencies and cell surface phenotypes of leukocytes compared to C57BL/6 mice. Frozen section analysis of liver revealed prevalent CD68 staining, indicative of a myeloid cell influx (Supplementary Fig. 2c, lower right). *Unc93b1*^3d/3d^*Pld3*^−/−^*Pld4*^−/−^ blood monocyte counts, though reduced compared to *Tlr9*^CpG11/CpG11^*Pld3*^−/−^*Pld4*^−/−^ mice, were still abnormally high compared to *Unc93b1*^3d/3d^*Pld4*^−/−^ or *Tlr9*^CpG11/CpG11^ mice (Fig. 1f). In the spleen, neutrophil frequencies were about 10-fold above wild-type levels though other myeloid subsets were similar to wild-type controls (Supplementary Fig. 5b, c). Total lymph node cell numbers in *Unc93b1*^3d/3d^*Pld3*^−/−^*Pld4*^−/−^ mice were significantly elevated over those of wild-type mice, mainly owing to an increase in B cells (Fig. 1h, i). Analysis of *Unc93b1*^3d/3d^*Pld3*^−/−^*Pld4*^−/−^ serum cytokine concentration similarly showed a pattern of partial normalization, as IFN-γ, TNF, IL-10, IL-18, and CXCL1 were reduced close to normal levels, while MCP3 and CXCL10 remained high compared to wild-type mice (Supplementary Fig. 5d,e). Finally, we found that *Unc93b1*^3d/3d^*Pld3*^−/−^*Pld4*^−/−^ mice bred well, whereas *Tlr9*^CpG11/CpG11^*Pld3*^−/−^*Pld4*^−/−^ females failed to become visibly pregnant or to give birth.

**Fig. 2:**
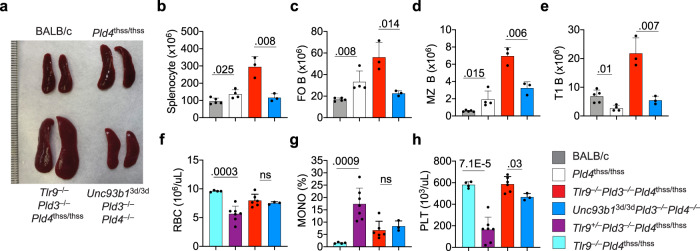
Splenomegaly and increased B cell numbers in *Tlr9*^−/−^*Pld3*^−/−^*Pld4*^thss/thss^ mice on the BALB/c background compared to *Unc93b1*^3d/3d^*Pld3*^−/−^*Pld4*^−/−^ mice. Mice of the indicated genotypes were compared for **a**, **b** splenomegaly, **c** splenic follicular B cells, **d** splenic MZ B cells, **e** splenic transitional-1 (T1) B cells, **f** blood erythrocytes, **g** blood monocytes, **h** platelets. Each data point indicates the value obtained with an independent mouse, bars show mean ± SD. In **b**–**e**, mouse groups were BALB/c (*n* = 5), *Pld4*^thss/thss^ (*n* = 4), *Tlr9*^−/−^*Pld3*^−/−^*Pld4*^thss/thss^ (*n* = 3), and *Unc93b1*^3d/3d^*Pld3*^−/−^*Pld4*^−/−^ (*n* = 3). In **f**–**h**, mouse strains compared were *Tlr9*^−/−^*Pld4*^thss/thss^ (*n* = 4), *Tlr9*^−/−^*Pld3*^−/−^*Pld4*^thss/thss^ (7), *Unc93b1*^3d/3d^*Pld3*^−/−^*Pld4*^−/−^ (*n* = 3), and *Tlr9*^+/–^*Pld3*^−/−^*Pld4*^thss/thss^ (*n* = 7). Statistical test was two-tailed *T* Test of the indicated pairwise group comparisons. Experiment was carried out once.

The *Tlr9*^CpG11^ allele was considered a null mutation that might have the added advantage of preventing compensatory upregulation of TLR7 signaling. However, the *Tlr9*^CpG11^ allele is likely a hypomorph, rather than a null mutation, particularly to phosphorothioate-linked ligands (D. Kono, personal communication, Supplementary Fig. 6). To rule out residual TLR9 activity, we subsequently generated *Tlr9*^−/−^*Pld3*^−/−^*Pld4*^thss/thss^ mice with a targeted *Tlr9* knockout and a spontaneous *Pld4* null mutation (thss), available on a BALB/c background. Comparison of *Tlr9*^−/−^*Pld3*^−/−^*Pld4*^thss/thss^ mice to *Tlr9*^+/–^*Pld3*^−/−^*Pld4*^thss/thss^ littermates and to *Unc93b1*^3d/3d^*Pld3*^−/−^*Pld4*^−/−^ mice revealed that complete TLR9 deficiency significantly ameliorated some abnormalities caused by PLD3/4 deficiency, such as thrombocytopenia. However, it failed to reverse other phenotypes or did so to a lesser extent than did the introduction of the *Unc93b1*^3d/3d^ mutation. Notably, *Tlr9*^−/−^*Pld3*^−/−^*Pld4*^thss/thss^ mice had more severe splenomegaly and B cell abnormalities than did *Unc93b1*^3d/3d^*Pld3*^−/−^*Pld4*^−/−^ mice (Fig. 2). As the 3d mutation blocks endosomal TLR signaling and the phenotype of *Unc93b1*^3d/3d^*Pld3*^−/−^*Pld4*^−/−^ mice was milder than that of *Tlr9*^−/−^*Pld3*^−/−^*Pld4*^thss/thss^ mice (or *Tlr9*^CpG11/CpG11^*Pld3*^−/−^*Pld4*^−/−^ mice), collectively, the results suggested that endolysosomal TLR sensing of RNA contributes significantly to the pathology caused by deficiency in both PLD3 and PLD4.

Independent evidence supporting the notion that exonuclease activity suppresses TLR7 autorecognition of RNA came from the B6.*Sle1Y*^aa^ mouse model, in which a twofold increase in *Tlr7* gene dosage in male mice drives spontaneous lupus nephritis^35^. Here, *Pld4* co-deficiency alone led to a greatly reduced birth rate and premature death of *B6.Sle1*^*Yaa*^*Pld4*^*−/−*^ compared to PLD4-sufficient littermates (Supplementary Fig. 7a, b). Conversely, lack of TLR7 extended the lifespan of *Tlr9*^+/CpG11^*Pld3*^−/−^*Pld4*^−/−^ mice, where the inflammation is triggered by a deficiency in PLD3 and PLD4 (Supplementary Fig. 7c). We conclude that TLR7 autorecognition of RNA is limited by PLD3 and PLD4.

### PLD3 and PLD4 are ssRNA exonucleases

**Fig. 3:**
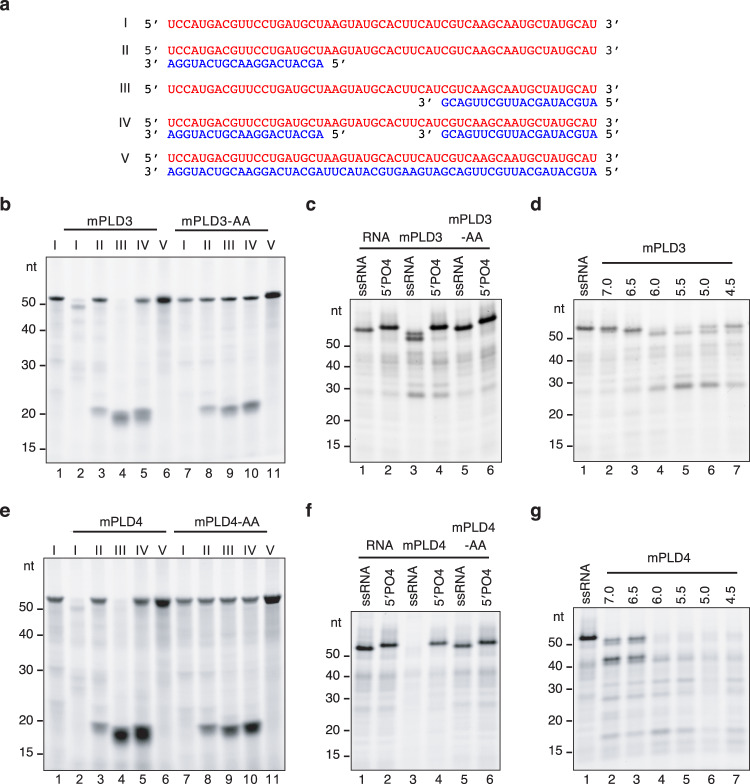
RNAse activity of PLD3 and PLD4 analyzed on oligonucleotide substrates. **a** Nucleotide sequences and schematic structures of substrates (I-V) used. The red strand is identical to “I” in all substrates and the blue strands are partly or completely complementary, annealed strands. **b** Denaturing Tris-Borate-EDTA PAGE and SYBR gold visualization of the products of incubation of mouse soluble recombinant PLD3 or enzymatically dead mutant PLD3-AA with the RNA substrates I-V, as indicated above the lanes. **c** Products of substrate I with or without phosphorylation at the 5′ end (5′PO_4_) digested by mouse PLD3 or PLD3-AA as in b. **d** PAGE analysis of products of incubation of substrate “I” as in (**b**), but with buffers adjusted to a pH of 4.5-7.0. **e**–**g** Digestion of RNA by mouse soluble recombinant PLD4 or PLD4-AA control enzyme. These experiments were carried out at least twice with similar results.

The overt pathology in *Tlr9*^CpG11/Cpg11^*Pld3*^−/−^*Pld4*^−/−^ mice and amelioration in *Unc93b1*^3d/3d^*Pld3*^−/−^*Pld4*^−/−^ mice suggested that PLD3 and PLD4 might degrade RNA. To directly assess this, we carried out the digestion of synthetic RNA substrates. First, recombinant soluble mouse PLD3 was generated and tested for nuclease activity on the RNA substrates shown in Fig. 3a. PLD3 had 5′-to-3′, but not 3′-to-5′, ssRNA exonuclease activity and lacked endonuclease activity (Fig. 3b, lanes 2–6). PLD3 protein carrying H-to-A mutations in both HxKxxxxD/E (HKD) motifs (PLD3-AA) lacked detectable enzymatic activity (lanes 7-11). Digestion was blocked by 5′ phosphorylation and was most efficient at pH 5.5 (Fig. 3c, d). Recombinant soluble mouse PLD4 showed similar RNAse activity in these assays but with a pH optimum of 5.0 and slightly slower kinetics than PLD3 (Fig. 3e–g). Recombinant human PLD3 and PLD4 showed strikingly similar features to their mouse counterparts in these assays (Supplementary Fig. 8). Thus, both PLD4 and PLD3 have 5′ ssRNA exonuclease activity, explaining their functional redundancy and suggesting that impaired degradation of both ssDNAs and ssRNAs might promote endosomal TLR signaling with attendant inflammation in mice deficient in both PLD3 and PLD4.

### RNA substrate specificity

**Fig. 4:**
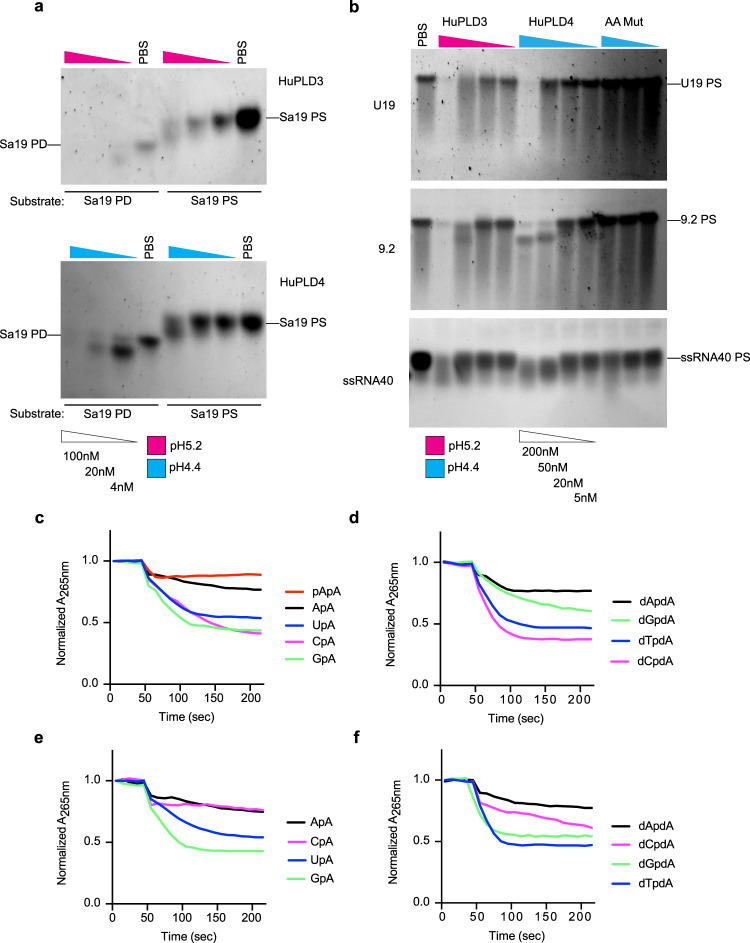
Specificity of RNAse activity of human PLD3 and PLD4. Analysis of digestion of **a, b** oligoribonucleotides or **c**–**f** dinucleotides. In **a**, substrates were Sa19 phosphodiester (5′-GGACGGAAAGACCCCGUGG-3′) (Sa19 PD) or its phosphorothioate form (Sa19 PS). In **b**, substrates were the following phosphorothioate-linked sequences: U_19_, UUUUUUUUUUUUUUUUUUU; 9.2, AGCUUAACCUGUCCUUCAA; ssRNA40, GCCCGUCUGUUGUGUGACUC. The concentration of enzyme and pH of reaction conditions are indicated. Oligonucleotides were at 2 μM. **c**–**f** Dinucleotide assay^68^ incubated the indicated ribodinucleotide (**c**, **e**) or deoxydinucleotide (**d**, **f**) substrates at 40 μM with 25 nM of PLD3 (**c**, d) or PLD4 (**e**, **f**) and followed the loss of adenosine absorbance in a linked assay. Experiments in (**a**, **b**) were repeated twice. Experiments in (**c**–**f**) were carried out at a range of concentrations as depicted in Supplementary Fig. 10.

In addition to the 55mer ssRNA (“I”, Fig. 3), PLD3 and PLD4 could digest other complex substrates to completion (Fig. 4a, b, Supplementary Fig. 9), indicating that all four ribonucleotides could be cleaved. However, “banding” patterns noted in partial digests suggested differences in efficiency. Phosphorothioate-linked oligoribonucleotides could be similarly digested, but more slowly than phosphodiester RNA of identical sequence (Fig. 4a, Supplementary Fig. 9). A dinucleotide digestion assay of ApA, GpA, CpA, and UpA, revealed differences in digestion efficiency, with CpA cleaved least efficiently by PLD4 (GpA > UpA > ApA > CpA) and ApA cleaved least efficiently by PLD3 (GpA > UpA > CpA > ApA) (Fig. 4c, e). Digestion of DNA dinucleotides showed an overall similar efficiency with a distinct pattern of cleavage preferences (Fig. 4d, f). As expected, a 5′ phosphorylated pApA dinucleotide was protected (Fig. 4c). Because of limitations of the substrate concentrations that can be used in the assay, accurate Km values could not be obtained for all substrates for PLD4, but for PLD3 estimated Km values in μM for GpA, UpA, ApA, and CpA were, respectively, 93, 84, 18, 72 (Supplementary Fig. 10).

### Short ssRNA fragments accumulate in *Pld3*^−/−^*Pld4*^−/−^ cells

**Fig. 5:**
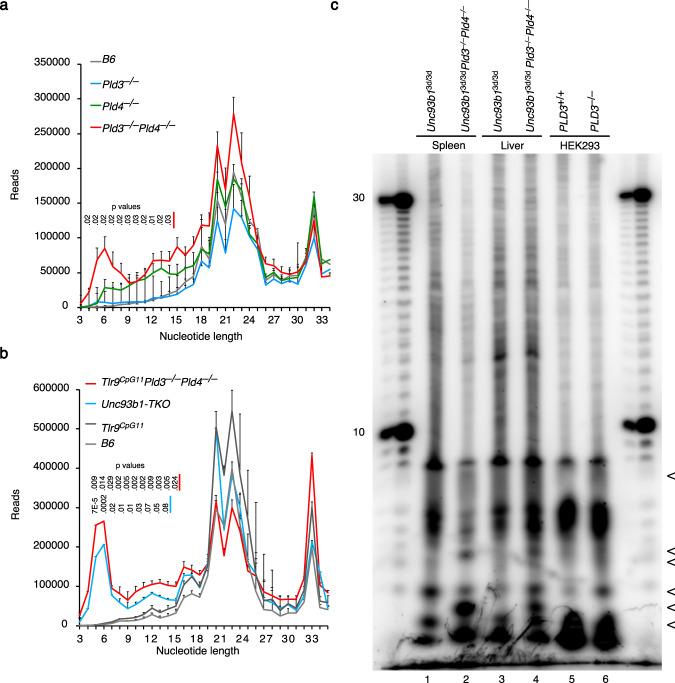
Accumulation of small nucleic acids in *Pld3*^−/−^*Pld4*^−/−^ and *Unc93b1*^3d/3d^*Pld3*^−/−^*Pld4*^−/−^ cells. Sequence length distributions of RNA isolated from the livers of **a** 2-week-old C57BL/6 (B6), *Pld3*^−/−^, *Pld4*^−/−^ or *Pld3*^−/−^*Pld4*^−/−^ mice, **b** Two-month-old B6, *Tlr9*^CpG11/CpG11^, *Tlr9*^CpG11/CpG11^*Pld3*^−/−^*Pld4*^−/−^ or *Unc93b1*^3d/3d^*Pld3*^−/−^*Pld4*^−/−^ mice. Shown are means and SEM of three mice/groups. In a, p values show a significant difference in *Pld3*^−/−^*Pld4*^−/−^ over C57BL/6 by unpaired two-tailed *T*-test. In (**b**), *p* values are shown for *Tlr9*^CpG11/CpG11^*Pld3*^−/−^*Pld4*^−/−^ or *Unc93b1*^3d/3d^*Pld3*^−/−^*Pld4*^−/−^ sequences, compared to C57BL/6 controls. Raw data for **a**, **b** are provided in Supplementary Tables 3 and 4. **c** Biochemical analysis of lysosome-associated nucleic acids. Lysosomal fractions were enriched from spleen or liver tissue of *Unc93b1*^3d/3d^*Pld3*^−/−^*Pld4*^−/−^ or *Unc93b1*^3d/3d^ mice, or from HEK^Blue-hTLR9^ cells that were PLD3-sufficient or -deficient, nucleic acids were 5′ labeled with [^32^P] phosphate using T4 polynucleotide kinase and electrophoresed on histidine-buffered 20% polyacrylamide gel. Radioactivity was revealed by phosphorimaging. Marker lanes show oligo dT lengths in nt. < symbol depicts the region of gel with differing abundance of nucleic acid signal. Sequence analysis shown in (**a**) and (**b**) was performed once (*n* = 3 independent mice/group). Lysosomal nucleic acid analysis was performed twice with similar results.

To assess whether nucleic acids are accumulating to drive the inflammation in the absence of PLD3 and PLD4, liver RNAseq data were “mined”. We first compared liver RNA from two-week-old C57BL/6, *Pld3*^*−/−*^, *Pld4*^*−/−*^ and *Pld3*^*−/−*^*Pld4*^*−/−*^ mice using a protocol that enriched miRNAs. Libraries were prepared for MiSeq analysis and then analyzed for the increased abundance of small RNAs that might be expected to build up in the absence of PLD3 and PLD4. The analysis revealed significantly enhanced signal at ~4–14 nt in all three *Pld3*^*−/−*^*Pld4*^*−/−*^ mice tested (Fig. 5a shows mean ± SEM). As inflammation itself might promote RNA fragmentation, we repeated this experiment comparing livers from two-month-old *Unc93b1*^3d/3d^*Pld3*^−/−^*Pld4*^−/−^ and *Tlr9*^CpG11/CpG11^*Pld3*^−/−^*Pld4*^−/−^ mice to those of age-matched wild-type and *Tlr9*^CpG11/CpG11^ liver samples. This analysis revealed a strikingly similar picture, with livers of both *Unc93b1*^3d/3d^*Pld3*^−/−^*Pld4*^−/−^ and *Tlr9*^CpG11/CpG11^*Pld3*^−/−^*Pld4*^−/−^ mice showing an increase in reads in the 4–12 nt range, with the greatest difference apparent at 5–6 nt (Fig. 5b). Finally, we compared length and abundance differences in nucleic acids isolated from lysosomes of spleen or liver cells of *Unc93b1*^3d/3d^*Pld3*^−/−^*Pld4*^−/−^ mice compared to *Unc93b1*^3d/3d^ controls by 5′-end-labeling with ^32^P-phosphate followed by polyacrylamide gel electrophoresis. The analysis revealed an increase in short fragments in the range of 2–6 nt in *Unc93b1*^3d/3d^*Pld3*^−/−^*Pld4*^−/−^ cells while fragments >15 nt appeared in similar abundance among the samples (Fig. 5c, lanes 1–4). A similar picture was seen in *PLD3*^−/−^ HEK293 cells compared to PLD3-sufficient HEK293 cells (lanes 5, 6). We conclude that very short fragments of nucleic acid accumulate in cells lacking both PLD3 and PLD4 and that the accumulation appears to be independent of the degree of immunopathology.

### ssRNA sensing by TLR7 and TLR13 in the absence of PLD3 and PLD4

**Fig. 6:**
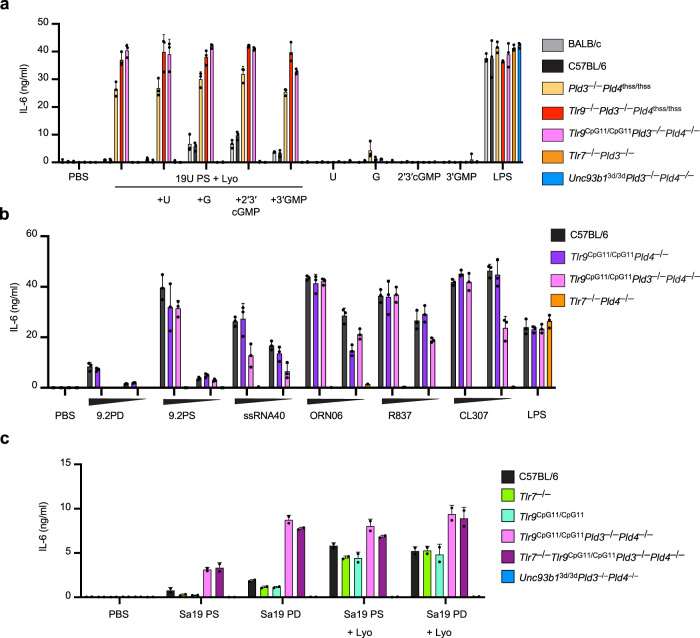
Analysis of RNA sensing by TLR7 and TLR13 in cells lacking PLD3 and PLD4. GM-CSF-elicited DCs of the indicated genotypes were challenged with the indicated ligands and assessed for IL-6 production. **a**, **b** Note the exaggerated IL-6 responses by PLD3/4-deficient cells to phosphorothioate-stabilized poly U (U_19_) but not to other TLR7 ligands. **c** Analysis of responses to TLR13 ligands Sa19-PD or Sa19-PS either given directly or packaged in Lyovec (+Lyo). In **a**, **b**, measurements were made in triplicate cultures. In **c**, duplicate cultures were analyzed. These results were repeated at least three times with similar results (also see Supplementary Fig. 11). All responses of *Pld3*^−/−^*Pld4*^thss/thss^, *Tlr9*^Cpg11/Cpg11^*Pld3*^−/−^*Pld4*^−/−^, and *Tlr9*^−/−^*Pld3*^−/−^*Pld4*^thss/thss^ DCs to U_19_ ligand exceeded wild-type control by significant margins (*p* < 0.001), whereas responses to 9.2-PD were significantly lower in *Tlr9*^Cpg11/Cpg11^*Pld3*^−/−^*Pld4*^−/−^ DCs compared to WT control (*p* < .001), as detailed in the reporting summary. Responses of *Tlr9*^Cpg11/Cpg11^*Pld3*^−/−^*Pld4*^−/−^ and *Tlr7*^−/−^*Tlr9*^Cpg11/Cpg11^*Pld3*^−/−^*Pld4*^−/−^ DCs were significantly higher than WT control to Sa19 PD (*p* = 0.003, *p* = 0.0006) and Sa19 PS (*p* = 0.01, *p* = 0.03), respectively.

To test the effect of PLD3/4 deficiency on responses to endosomal TLR RNA ligands, we challenged primary dendritic cells (DCs) with a variety of TLR7 ligands. Primary DCs expanded from *Tlr9*^CpG11/CpG11^*Pld3*^−/−^*Pld4*^−/−^, *Tlr9*^−/−^*Pld3*^−/−^*Pld4*^thss/thss^ and *Pld3*^−/−^*Pld4*^−/−^ bone marrow showed enhanced IL-6 secretion to U_19_, diminished response to ssRNA40 and 9.2-PD, and no change to ORN06 or 9.2-PS (Fig. 6a, b, Supplementary Fig. 11). Supplementation with nucleosides augmented the response to U_19_ of PLD3/4-sufficient but not PLD3/4-deficient DCs (Fig. 6a). Responses to nucleoside analogues R837 and CL307 were relatively unaffected (Fig. 6b). As expected, control *Tlr7*^−/−^*Pld3*^−/−^ and *Unc93b1*^3d/3d^*Pld3*^−/−^*Pld4*^−/−^ DCs produced no IL-6 to these TLR7 ligands, yet responded to the TLR4 agonist LPS.

To assess in the context of PLD3/4 deficiency the response to TLR13, a sensor of bacterial rRNA^5,8^, we compared responses to the TLR13 ligand Sa19-PD and its phosphorothioate-linked derivative Sa19-PS. Primary DCs expanded from *Tlr9*^CpG11/CpG11^*Pld3*^−/−^*Pld4*^−/−^ or *Tlr7*^−/−^*Tlr9*^CpG11/CpG11^*Pld3*^−/−^*Pld4*^−/−^ bone marrow cultures under GM-CSF conditions showed enhanced responses to Sa19, particularly when the ligand was given in the absence of the transfection reagent Lyovec (Fig. 6c). Notably, *Unc93b1*^3d/3d^*Pld3*^−/−^*Pld4*^−/−^ cells failed to respond. These data indicate that PLD3 and PLD4 limit TLR13 activation in primary DCs. A similar sensitization to Sa19 was seen in PLD3-deficient compared to unmutated HEK293^Blue-Tlr13^ indicator cells (Supplementary Fig. 12). We conclude that *Unc93b1*-dependent RNA sensing promotes inflammation in *Tlr9*^*−/−*^*Pld3*^−/−^*Pld4*^−/−^ mice, that PLD3 and PLD4 may regulate responses to a subset of TLR7 ligands, and that TLR7 and TLR13 responses are elevated in some cell types, however TLR7 signaling is not dominant in driving disease in the absence of PLD3 and PLD4.

### Type I IFN signature in *Unc93b1*^*3d/3d*^*Pld3*^*−/−*^*Pld4*^*−/−*^ mice

**Fig. 7:**
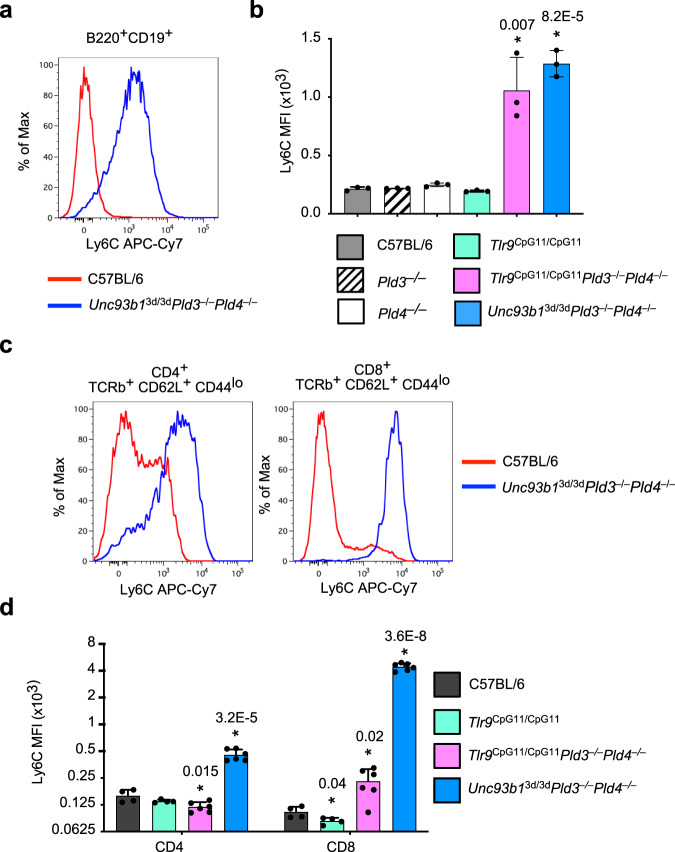
Elevated Ly6C on *Unc93b1*^3d/3d^*Pld3*^−/−^*Pld4*^−/−^ B and T cells. Analysis of Ly6C upregulation on lymphocyte subsets in lymph nodes of mouse strains of the indicated genotypes. **a**, **b** Elevated Ly6C on B cells of *Unc93b1*^3d/3d^*Pld3*^−/−^*Pld4*^−/−^ and *Tlr9*^CpG11/CpG11^*Pld3*^−/−^*Pld4*^−/−^ mice. **c**, **d** Comparison of Ly6C expression on CD4^+^ T or CD8^+^ T cells from pooled lymph nodes of the indicated strains. Bar graphs show mean ± SD with each symbol representing an individual mouse. In **b**, *n* = 3 independent mice/group. In d, groups were composed of independent mice of the following genotypes: C57BL/6 (*n* = 4), *Tlr9*^CpG11/CpG11^ (*n* = 4), *Tlr9*^CpG11/CpG11^*Pld3*^−/−^*Pld4*^−/−^ (*n* = 6), and *Unc93b1*^3d/3d^*Pld3*^−/−^*Pld4*^−/−^ (*n* = 6). Unpaired two-tailed *T*-tests were performed by comparing groups to C57BL/6; asterisks indicate significant differences with *p* values given. These experiments were performed twice with similar results.

Importantly, *Unc93b1*^3d/3d^*Pld3*^−/−^*Pld4*^−/−^ mice showed phenotypic changes consistent with type I IFN-driven activation. Flow cytometry analysis of *Unc93b1*^3d/3d^*Pld3*^−/−^*Pld4*^−/−^ spleen and lymph node B cells revealed extraordinarily high expression of surface Ly6C (Fig. 7a), a phenotype that was shared with *Tlr9*^CpG11/CpG11^*Pld3*^−/−^*Pld4*^−/−^ mice but not with *Pld3*^−/−^ or *Pld4*^−/−^ strain controls (Fig. 7b). In addition, CD4^+^ and CD8^+^ T cells from *Unc93b1*^3d/3d^*Pld3*^−/−^*Pld4*^−/−^ mice showed elevated Ly6C expression (Fig. 7c, d). Expression of Ly6C on B and T cells is known to be driven by type I IFN and by IFN-γ^36^ but because B cells from *Pld4*^−/−^ mice lacked Ly6C upregulation despite their elevated serum IFN-γ concentration, the results implicated type I IFN. When wild-type spleen cells were transferred into *Unc93b1*^3d/3d^*Pld3*^−/−^*Pld4*^−/−^ mice, the incoming B and T cells upregulated Ly6C; however, when the transferred cells were mutated in the type I IFN receptor, *Ifnar1*, no such upregulation occurred (Supplementary Fig. 13). Although we were unable to directly detect type I IFN in their sera, these data suggested that type I IFN is constitutively overproduced in both *Unc93b1*^3d/3d^*Pld3*^−/−^*Pld4*^−/−^ and *Tlr9*^CpG11/CpG11^*Pld3*^−/−^*Pld4*^−/−^ mice.

The ameliorated disease and IFN signature in *Unc93b1*^3d/3d^*Pld3*^−/−^*Pld4*^−/−^ compared to *Tlr9*^CpG11/CpG11^*Pld3*^−/−^*Pld4*^−/−^ mice was supported by independent RNA sequencing (RNAseq) analysis of liver tissue (summarized in Supplementary Fig. 14 and in more detail in Supplementary Data 1). Overall, the data indicated that RNA sensing by endosomal TLRs contributed significantly to the pathogenesis of *Pld3*^−/−^*Pld4*^−/−^ mice. However, inflammation was not fully normalized in the absence of TLR9, TLR7, or all endosomal TLR signaling. The striking difference in phenotype and liver RNA expression between *Tlr9*^*CpG11/CpG11*^*Pld3*^−/−^*Pld4*^−/−^ and *Unc93b1*^3d/3d^*Pld3*^−/−^*Pld4*^−/−^ mice leads us to conclude that RNA sensing by endosomal TLRs promotes inflammation in this model. The apparent production of, and triggering by, type I IFN in *Unc93b1*^3d/3d^*Pld3*^−/−^*Pld4*^−/−^ mice suggests that non-TLR nucleic acid sensors were also activated.

### The type I IFN-inducing pathway in *Unc93b1*^3d/3d^*Pld3*^−/−^*Pld4*^−/−^ mice requires STING

**Fig. 8:**
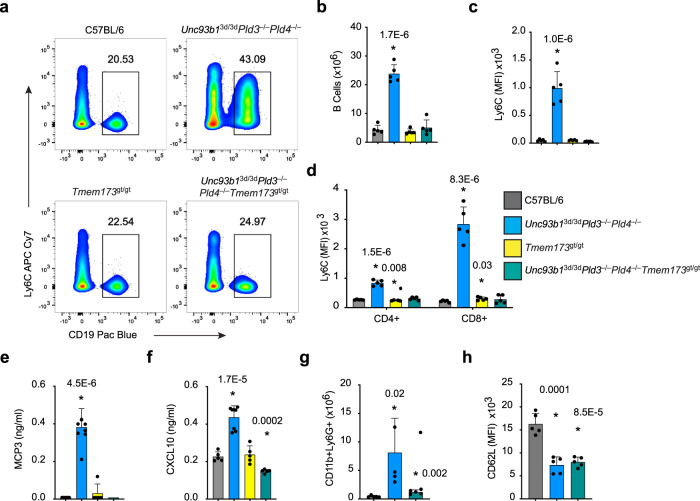
STING-driven increase of Ly6C, MCP3, CXCL10, and neutrophils in *Unc93b1*^3d/3d^*Pld3*^−/−^*Pld4*^−/−^ mice. Mice of the indicated genotypes were assessed for the expression levels of Ly6C on B and T cells from lymph nodes. **a**–**c** Analysis of B cells. **d** Ly6C levels on CD4^+^ and CD8^+^ T cells. Analysis of concentration of **e** MCP3 and **f** CXCL10 in sera of indicated mice. Splenic neutrophils were assessed for **g** number and **h** surface expression of CD62L. Bar graphs show mean ± SD with each symbol representing an individual mouse. Unpaired two-tailed *T*-tests were performed by comparing groups to C57BL/6. These experiments were performed at least twice with similar results. In most experiments, five independent mice were analyzed per group, except for **e**, **f**, where eight mice were in the *Unc93b1*^3d/3d^*Pld3*^−/−^*Pld4*^−/−^ group.

Because *Unc93b1*^3d/3d^*Pld3*^−/−^*Pld4*^−/−^ mice retained a striking IFN signature, we assessed the contribution of the stimulator of interferon genes (STING) by generating STING-deficient *Tmem173*^gt/gt^*Unc93b1*^3d/3d^*Pld3*^−/−^*Pld4*^−/−^ mice. In these quadruple mutants, we found that the elevated Ly6C expression on B and T cells was normalized to levels of C57BL/6 or *Tmem173*^gt/gt^ controls (Fig. 8a–d). Moreover, serum concentrations of the chemokines MCP3 and CXCL10 returned to wild-type levels (Fig. 8e, f). Splenic neutrophil numbers from *Tmem173*^gt/gt^*Unc93b1*^3d/3d^*Pld3*^−/−^*Pld4*^−/−^ mice were normalized compared to *Unc93b1*^3d/3d^*Pld3*^−/−^*Pld4*^−/−^ mice, however the lower expression of CD62L on these neutrophils compared to the wild type indicated abnormalities may still remain (Fig. 8g, h). Because many cytoplasmic nucleic acid sensing pathways converge through a pathway controlled by STING^37,38^, the results support a model in which excess nucleic acid fragments from endolysosomes lacking PLD3 and PLD4 trigger cytoplasmic sensors requiring STING.

## Discussion

Here we show that TLR responses to both DNA and RNA primarily drive the pathology in mice lacking both PLD3 and PLD4 nucleases. Rescued viability in TLR9-deficient *Pld3*^−/−^*Pld4*^−/−^ mice and in TLR9 antibody-treated *Unc93b1*^+/3d^*Pld3*^−/−^*Pld4*^−/−^ mice clearly supports a role for the ssDNA sensor TLR9. Previous studies showed that repeated challenge of mice with a TLR9 ligand is sufficient to induce a syndrome similar to HLH^39^ and that the disease that develops is partly dependent upon IFN-γ^40^. IFN-γ was similarly implicated in models of HLH triggered by viral infection in mice mutated in perforin, *Rab27a* or *Unc13d*^41–44^. But even in these models, the MyD88 pathway appeared to be more important than IFN-γ^42,45,46^. Moreover, patients have been identified who lack functional IFN-γ receptor yet develop HLH^47^. In *Pld3*^−/−^*Pld4*^−/−^ mice, TLR9 was clearly not the sole driver of disease; IFN-γ contributed to, but was dispensable for, premature death. That disease was further ameliorated in *Unc93b1*^3d/3d^*Pld3*^−/−^*Pld4*^−/−^ mice was consistent with a contribution of an endolysosomal TLR RNA sensor. Residual abnormalities and type I interferon production in *Unc93b1*^3d/3d^*Pld3*^−/−^*Pld4*^−/−^ mice were normalized in *Tmem173*^gt/gt^*Unc93b1*^3d/3d^*Pld3*^−/−^*Pld4*^−/−^ mice, indicating that STING or an associated cytoplasmic nucleic acid sensor was also chronically triggered in the absence of PLD3 and PLD4. We show for the first time that PLD3 and PLD4 are ssRNA exonucleases and that short oligonucleotides accumulate in tissues of mice lacking both PLD3 and PLD4. Consistent with this, PLD3 is likely to be identical to classical spleen acid exonuclease^30^, an enzyme that degrades both ssDNA and ssRNA^48^. As endocytosed ssDNA is degraded more slowly in cells lacking PLD3 and PLD4^30^, it is likely that the accumulation of both ssDNA and ssRNA fragments in these mice triggers the inflammation. Importantly, small fragments of ssDNA (3-5 nt) can augment TLR9 activation in cells and in biochemical assays^49,50^; similarly, TLR7, human TLR8, and mouse TLR13 are also activated by RNA degradation products, including nucleosides in the cases of TLR7 and hTLR8^6–8^. We show that TLR13 ligands and TLR7 ligands are degraded by PLD3 and PLD4 and that the recognition by DCs of TLR13 ligands and a subset of TLR7 ligands is sensitized in the absence of PLD3 and PLD4. Besides the endosomal TLRs, no other nucleic acid sensors are known to recognize such short (2-10 nt) ssRNA or ssDNA fragments. Our results suggest that STING-coupled sensing is also triggered. Overall, our studies underscore the likelihood that PLD3 and PLD4 are major nucleases critical in preventing the triggering of multiple RNA and DNA receptors.

Our results may have clinical implications, as the treatments for HLH and MAS have not been firmly established^51^. Among therapies being considered include the blockade of the cytokines IFN-γ, TNF, IL-6, IL-1 and IL-18. To the extent that the *Pld3*^−/−^*Pld4*^−/−^ mouse simulates human HLH or a particular subtype thereof, our studies indicate that the clinical benefit of blockade of IFN-γ might be modest compared to blockade of TLR9, or perhaps additional TLRs. Importantly, anti-TLR9-treated *Unc93b1*^+/3d^*Pld3*^−/−^*Pld4*^−/−^ mice showed a clinical benefit even though the disease was already severe prior to treatment, indicating that TLR9 blockade might be beneficial long after HLH disease initiation.

Unlike other mouse models of HLH, the *Pld3*^−/−^*Pld4*^−/−^ model is the only one available that develops disease spontaneously. Preclinical studies using this model may be easier to interpret than infection-driven models, as potential treatments in the latter models may affect microbe replication and levels independent of HLH inflammation; infection may also have confounding effects on the immune system through the pathogen’s ability to subvert particular pathways.

Although IFN-γ makes some contribution to disease, such as hepatic microvesicular steatosis, *Ifng*^*−/−*^*Pld3*^−/−^*Pld4*^−/−^ mice were not rescued from early death. IFN-γ has been shown to promote liver steatosis in mice after its induction by coinjection of IL-12 and IL-18^52^. HLH-like features were previously seen in a mouse model of IFN-γ overexpression^53^, including splenomegaly, lymphadenopathy, monocyte activation, and liver pathology, arguing that IFN-γ alone can drive considerable pathology. However, these features of HLH were retained in *Ifng*^*−/−*^*Pld3*^−/−^*Pld4*^−/−^ mice.

The striking differences in phenotype among *Pld3*^−/−^*Pld4*^−/−^, *Unc93b1*^3d/3d^*Pld3*^−/−^*Pld4*^−/−^ and TLR9-deficient *Pld3*^−/−^*Pld4*^−/−^ strains revealed that both TLR9 and RNA-sensing TLRs drive the disease caused by deficiency of PLD3 and PLD4. The activity of TLR9 in *Pld3*^−/−^*Pld4*^−/−^ mice was associated with a loss of B cells, which re-emerged and were abundant in *Tlr9*^*CpG11/CpG11*^*Pld3*^−/−^*Pld4*^−/−^ and *Unc93b1*^3d/3d^*Pld3*^−/−^*Pld4*^−/−^ mice. TLR9 signaling has been shown to be toxic to B cells in several contexts^54,55^. HLH is also associated with pancytopenia. Anti-TLR9-treated *Unc93b1*^+/3d^*Pld3*^−/−^*Pld4*^−/−^ mice showed significantly restored B and T cell numbers and mouse weight.

The TLRs responsible for RNA-driven inflammation in the *Pld3*^−/−^*Pld4*^−/−^ model are unknown, but likely include ssRNA sensors TLR7 and TLR13. TLR3 and TLR8 seem less likely to contribute to inflammation in this model because PLD3 and PLD4 do not degrade the dsRNA ligands of TLR3, and TLR8 seems to have the negligible proinflammatory functional capacity in the mouse. Although TLR13 sees bacterial ribosomal RNA, it may also recognize some small fraction of self-RNA carrying a similar sequence. Further genetic breeding studies should clarify this.

PLD3 and PLD4 could digest in vitro dinucleotides and ssRNAs of diverse sequences, albeit with a range of efficiencies. Sa19 digestion is predicted to destroy its ability to stimulate TLR13, explaining the enhanced responses of PLD3/4 deficient DCs to that ligand. However, the effects of PLD3/4 deficiency on the TLR7 response were more complex, as we saw the selective enhancement of responses to U_19_ but not to other TLR7 agonists tested (Fig. 6). Indeed, responses to ligands ssRNA40 and 9.2-PD appeared to be lower in the absence of PLD3 and PLD4, and responses to ORN06 and 9.2-PS were unchanged. Because TLR7 (and TLR8) oligoribonucleotide ligands require significant nuclease processing to generate stimulatory mixtures of nucleosides and short oligoribonucleotides to occupy the two binding sites^56–58^, PLD3 and PLD4 might both destroy and create stimulatory fragments, depending upon the initial ssRNA sequence. We previously showed that digestion by PLD3 and PLD4 of the TLR9 agonist 2216 is blocked by its secondary structure, requiring prior endonuclease cleavage, likely by DNase2, to reveal their in vivo effects on responsiveness^30^. Similarly, in vivo degradation of particular ssRNA substrates by PLD3 and PLD4 is likely limited by prior endonuclease processing and secondary structure in addition to the primary sequence. It is notable in this regard that RNaseT2 and RNase A family endonucleases generate ssRNA fragments with 5′ hydroxyl ends required for digestion by PLD3 and PLD4^7,59,60^. So, individual RNAs likely differ in their requirements for processing by endo and exonucleases for recognition by TLR7/8, which in turn could affect the timing or duration of signaling during endolysosome acidification and maturation.

Several observations indicated that one or more STING-coupled sensors of nucleic acid is triggered in mice deficient in PLD3 and PLD4, leading to the production of type I IFN. *Unc93b1*^3d/3d^*Pld3*^−/−^*Pld4*^−/−^ B cells express Ly6C, which is normally not expressed on B cells. Owing to IFN response elements in its promoter, Ly6C can be upregulated in B cells by IFN-α/β, IFN-γ or in vivo challenge with TLR9 ligand^36,61^. As IFN-γ is high in sera of *Pld4*^−/−^ mice, which lack B cell expression of Ly6C, we infer that type I IFNs are elevated and drive Ly6C expression in *Unc93b1*^3d/3d^*Pld3*^−/−^*Pld4*^−/−^ B cells. High serum levels of CXCL10 were found in *Ifng*^*−/−*^*Pld3*^−/−^*Pld4*^−/−^ and *Unc93b1*^3d/3d^*Pld3*^−/−^*Pld4*^−/−^ mice. *Tmem173*^gt/gt^*Unc93b1*^3d/3d^*Pld3*^−/−^*Pld4*^−/−^ mice lost this IFN signature and the production of CXCL10 and MCP3, demonstrating that the STING cytosolic nucleic acid sensing pathway was triggered. It is currently unclear which sensors might be involved because none of the currently described nucleic acid sensors are known to recognize the short single-stranded nucleic acid structures that accumulate in *Unc93b1*^3d/3d^*Pld3*^−/−^*Pld4*^−/−^ mice. However, 12 bp dsDNA with protruding single-stranded Gs (Y-form DNA) has been shown to be able to directly activate cGAS^62^. It is also unclear how lysosomal nucleic acids might find their way to the cytoplasmic sensors. Neuronal lysosomes of *Pld3*^−/−^ mice were considerably larger and denser^63^, and perhaps are less stable. A build-up of oligonucleotides in lysosomes might be released by rupture, pass through the lysosomal membrane nonspecifically, or be transported by a protein or pore.

## Methods

### Mice

All animal studies were approved by The Scripps Research Institute (TSRI) Institutional Animal Care and Use Committee. Mice were housed in specific pathogen-free conditions at 68–72 °F and 30–70% humidity, with 6pm–6am nocturnal dark/light cycle. C57BL/6J mice were obtained from Jackson Labs and bred at TSRI. *Pld3*^−/−^ were generated by CRISPR mutagenesis in our laboratory on the C57BL/6 J background^30^. *Pld4*^−/−^ was originally generated on the 129/J strain and extensively backcrossed to C57BL/6J^30^. The following mouse strains have been described previously and were obtained from Jackson Laboratories on the C57BL/6 J background: *Ifng*^−/−^; *Ifnar1*^−/−^, *Tmem173*^gt/gt^, *Tlr7*^−/−^. B6.Cg-*Sle1*^*NZM2410/Aeg*^ *Yaa*/DcrJ mice (named B6.Sle1^Yaa^ in this study) are on the C57BL/6 background but have elements from NZM2410 Chr 1 and the Yaa chromosome from BXSB strain. C57BL/6J-*Tlr9*^*M7Btlr*^/Mmjax (named *Tlr9*^*CpG11*^ in this manuscript) is a point missense mutant generated on the C57BL/6 J background. BALB/cJ-*Pld4*^*thss*^/GrsrJ is a spontaneous *Pld4* mutant identified on the BALB/c background. *Unc93b1*^3d/3d^ mice^32^ (C57BL/6J background) were kindly provided by B. Beutler, Univ. Texas Southwestern with assistance from D. Kono, TSRI. *Tlr9*^−/−^ on the BALB/c background was kindly provided by Ann Marshak-Rothstein^64^.

### Genotyping

Interbred mice were identified by PCR genotyping using DNA from tail clip biopsy. Methods for *Pld3* and *Pld4* mutant genotyping have been described^30^. Oligonucleotide primers used to PCR genotype the following genes, *Unc93b1*, *Tlr7* WT, *Tlr7* KO, *Tmem173*^gt^, BALB/c *Tlr9*^−/−^, *Ifng*^−/−^, *Pld4*^*ths*s^ and *Tlr9*^CpG11^ are listed in Supplementary Table 2. To distinguish between the WT and mutant alleles, the following enzymes were used to digest the PCR products. *Tmem173*^gt^, BtsC1; *Unc93b1*^3d/3d^, BstAPI; *Pld3*, Box1; *Pld4*^thss^, HpyCH4III. The *Tlr9*^CpG11^ allele was confirmed by sequencing of the PCR amplicon.

### Blood analysis

Erythrocyte and platelet counts, and monocyte percentages in heparinized whole blood from age-matched male and female animals were measured using the IDEX Procyte DX Machine for blood subset analysis. Plasma or serum diluted 1:1 was used to measure the levels of cytokines depicted in Supplementary Fig. 5, which were measured using FlowCytomix beads (eBioscience) with analysis on a LSRII flow cytometer (BD Bioscience). IL-18 levels were measured by analyzing sera diluted 1:5 by ELISA (MBL). MCP3 and CXCL10 levels in Fig. 8e, f were measured by ELISA (Invitrogen) according to manufacturer directions.

### Flow cytometry analysis of leukocyte compartments

Tissue suspensions were generated by mashing spleen and lymph nodes between frosted glass slides. Red blood cells were lysed with ACK buffer and cells filtered to generate single-cell suspensions. Cells were pre-incubated with Ghost Violet 510 (Tonbo bioscience) for 30 min to identify dead cells before addition of 1 μg/10^6^ cells of Fc Block (2.4G2) prior to staining with antibodies at a final concentration of 1/200. Clone numbers of antibodies used and their suppliers are indicated in Supplementary Table 1. Splenic natural killer (NK) cell proportions were measured by flow cytometry of splenocytes from age and sex-matched animals using the following gates (TCRβ^−^ CD49b^+^). The following gating strategies were used to identify and quantify different leukocyte subsets. NKT (TCRβ^+^CD49b^+^); Naive CD4^+^ (TCRβ^+^ CD4^+^CD8a^−^CD62L^+^CD44^lo^); Effector CD4^+^ (TCRβ^+^ CD4^+^CD8a^−^CD62L^lo^CD44^+^); Naive CD8^+^ (TCRβ^+^ CD4^−^CD8α^+^CD62L^+^CD44^lo^); Effector CD8^+^ (TCRβ^+^ CD4^−^CD8α^+^CD62L^lo^CD44^+^); B cells (CD19^+^B220^+^) or (CD45.2^+^B220^+^CD93^-^MHCII^+^); Follicular B cells (CD19^+^B220^+^CD93^−^IgM^+^CD23^+^); Marginal zone B cells (MZ) (CD19^+^B220^+^CD93^−^CD23^lo^IgM^+^CD21^hi^); Transitional 1 B cells (T1) (B220^+^CD93^+^CD23^lo^IgM^+^); Neutrophils (CD11b^+^Ly6G^+^SSC^med^). Hemophagocytosis was identified by flow cytometry by gating splenic cells as follows (CD45^+^CD19^−^CD11b^+^CD11c^+^Ter119^+^). A summary of these gating strategies is provided in Supplementary Fig. 15.

### CD68+ liver frozen section stains

Livers from mice 2 or 12 weeks of age and sex matched were frozen in optimal cutting temperature (O.C.T., Sakura). Then 7 μm sections were cut, fixed with acetone for 10 min, and stained with anti-CD68 Alexa Fluor 647 (FA-11, Biolegend). After washing in PBS, nuclei were stained with Hoechst 33258 for 5 min prior to mounting in Prolong Diamond Antifade mounting medium (Invitrogen). Sections were imaged using the Keyence BZ-X710 fluorescence microscope. Images were analyzed using Image J vers 2.0.0-rc-65/1.51n.

#### Dendritic cell generation and stimulation

BMDCs were expanded in vitro from bone marrow precursors in IMDM media (Lonza) containing 10% FBS, 2 mM Glutamine, Penicillin and Streptomycin and recombinant murine GM-CSF (Peprotech) at 10 ng/ml, with media changes every three days. Cells were plated at a density of 10^6^/ml in round-bottom plates on day 8 in media lacking GM-CSF. Stimuli were added in serum-free media, and supernatants were collected 18–24 h later for cytokine analyses. ssRNA40 and ORN06 were purchased from InvivoGen pre-complexed in Lyovec and used at concentrations beginning at 4 μg/ml. The following compounds were purchased from InvivoGen and used at the following starting concentrations, Sa19 PS [0.1 μM], R837 [5 μg/ml], R848 [0.5 μg/ml], CL307 [2.8 μg/ml], LPS [100 ng/ml], VACV70 and ISD were complexed with Lyovec and used at 1 μg/ml. Oligoribonucleotides purchased from IDT included phosphodiester Sa19-PD [2 μM], phosphorothioate Sa19-PS [0.5 μM], 9.2 PD [1.4  μM], 19U PS [1.4 μM]. 9.2 PD was complexed using pLArg (Sigma) at a ratio of 1.6 μg RNA to 2.8 μg pLArg. 9.2 PS was complexed using Lyovec. Oligonucleotides purchased from IDT included A3’13-PS [0.5 μM]. Sixfold dilutions of compounds were used when depicted as triangles. When added, guanosine (G) (Sigma), uridine (U) (Sigma), 2′-3′ cGMP (Biolog Life Science Institute) or 3′GMP (Biolog Life Science Institute) were added directly to the culture at 250 μM.

#### Dinucleotide substrate assay

Enzymatic activity assay on dinucleotide substrates was performed as described^65^. Adenosine deaminase (ADA) purchased from Worthington was resuspended in 1 ml water then diluted to 20 μg/ml in 0.5× PBS. The dinucleotide substrates GpA, ApA, pApA, UpA, CpA, and the DNA equivalents, dGpdA, dApdA, dCpdA, and dTpdA were purchased from TriLink Biotechnologies and reconstituted in RNase-free water. The final reaction condition for PLD3 and PLD4 was 50 mM MES pH 6.1, 20 mM NaCl. Each reaction consisted of dinucleotide substrate (ranging from 5 to 100 μM), ADA enzyme (2  μg/ml), and either Human PLD3 (25 nM) or Human PLD4 (25 nM) in a total volume of 250 μL. The reaction was performed with the Nanodrop 3000 C in a heated quartz cuvette (37 °C) and absorbance at 265 nm measured every 15 s. The tested enzyme was added after four initial absorbance readings (1 min) and absorbance measured for another 5 min. In the absence of an added enzyme, such as substrate alone, or ADA alone, no change in absorbance was observed throughout the 6 min of recording. In some cases, absorbance was normalized between substrates by dividing absorbance at all time points for a given substrate by that measured for that substrate at 10 s.

### RNA sequencing and analysis

Total RNA was isolated from liver tissue fragments in Trizol Reagent (Thermo) according to the manufacturer’s instructions. RNA was prepared into RNAseq libraries using the NEBNext Ultra Directional RNA Library Prep Kit for Illumina (NEB, Cat. #E7760) following the manufacturer’s recommended protocol. The libraries were then PCR amplified 15 cycles using barcoded PCR primers, purified and size selected using AMPure XP Beads before loading onto an Illumina NextSeq500 for 75 base single read sequencing. A total of >20 million “passed filter” reads were obtained for each of the samples.

### Statistical analyses

The expression levels of mouse transcripts (Supplementary Fig. 14) from 2-week-old livers were estimated using Salmon. Mouse GENCODE gene set (release M10) was used as transcriptome annotation. The transcript abundances were converted into gene-level read counts by using the tximport package. Analysis of differential gene expression was carried out using the program DEGSeq2. For 2-month-old livers, reads were aligned to mouse genome build GRCm38 using STAR v2.5.2a and gene abundance was estimated using HTSeq Count v0.11.0. Raw gene counts were then used to get differentially expressed genes using Bioconductor package DESeq2 v1.20.0. The differentially expressed genes (DEGs) were identified as those with false-discovery rates <0.05, absolute fold change >2. Visualizations and clustering analysis were carried out using Heatmapper. Other data comparisons used the program Prism (Graph Pad), with Bonferroni’s correction for multiple comparisons. When two groups were compared, an unpaired, two-tailed *T* test was used.

### Analysis of short RNAs in liver by RNAseq

For each sample, ~1 μg total RNA was treated in a series of enzymatic reactions that take place in a successive manner without isolation or purification of desired products. First, the sample was reacted with 10 U of T4 polynucleotide kinase (New England Biolabs) containing 70 mM Tris-HCl, pH 7.6, 10 mM MgCl_2_, 5 mM DTT, and 1 mM ATP in a total volume of 10 µL at 37 °C for 30 min to phosphorylate the 5′ ends of the RNAs and remove any 3′ phosphoryl groups. The reaction was then incubated at 70 °C for 10 min to inactivate the kinase. Then, 10 pmol of an Illumina 5′ adenylated DNA p7 adapter (AppTGGAATTCTCGGGTGCCAAGG-C3 spacer) was ligated to all available 3′ ends in the RNA sample with 400 U of T4 RNA Ligase 2, truncated (New England Biolabs), in a reaction containing 85 mM Tris-HCl, pH 7.5, 15 mM MgCl_2_, 3.5 mM DTT, 10% PEG8000, 40 U Ribonuclease inhibitor (ThermoFisher Scientific) at 25 °C for 1 h in a 20 μL final volume. 20 pmol of reverse transcription (RT) primer GCCTTGGCACCCGAGAATTCCA was annealed to the ligated and unligated p7 adapter in a 25 μL final volume. Next, 25 pmol of a p5 RNA adapter sequence (GUUCAGAGUUCUACAGUCCGACGAUC (RNA)) was ligated to the 5′ ends of the RNA sample with 15 U of T4 RNA Ligase 1 (New England Biolabs), in a reaction containing 73.7 mM Tris-HCl, pH 7.5, 13.3 mM MgCl_2_, 2.6 mM DTT, 6.7% PEG8000, 40 U RNaseOUT Ribonuclease inhibitor (ThermoFisher Scientific) at 25 °C for 1 h in a 30 μL final volume. Then, a reverse transcription reaction containing 400 U of Superscript III (ThermoFisher Scientific) and 80.3 mM Tris-HCl, pH 8.3, 11.4 mM MgCl_2_, 4.4 mM DTT, 5% PEG8000, 37.5 mM KCl, 80 U RNaseOUT Ribonuclease inhibitor (ThermoFisher Scientific) and 0.5 mM dNTPs at 50 °C for 1 h in a 40 μL final volume. This was added directly to a PCR reaction containing 50 μL 2X KAPA HiFi HotStart ReadyMix and 62.5 pmol of each full-length p5 and p7-barcoded Illumina primers (AATGATACGGCGACCACCGAGATCTACACGTTCAGAGTTCTACAGTCCGA and CAAGCAGAAGACGGCATACGAGATNNNNNNGTGACTGGAGTTCCTTGGCACCCGAGAATTCCA (NNNNNN = 6 base barcode)) in a final volume of 100 μL. The reaction was performed as follows: initial hold 95 °C, 3 min; 12 cycles of 98 °C, 20 s; 62 °C, 30 s; 72 °C, 15 s; final hold 72 °C, 2 min. DNA libraries were isolated using AmpureXP bead (Beckman-Coulter) purification according to manufacturer protocol. Products were purified on 4% Egel-EX agarose gels and bands ~140–160 bp were excised from the gel using standard agarose gel extraction methods. Libraries were loaded onto a NextSeq500 and sequenced with 75 base single-end reads targeting 5 M reads per sample.

### Lysosome nucleic acid preparation and analysis

Lysosome containing fractions from liver, spleen, and HEK293^Blue-hTLR9^ cells were enriched using a kit (Thermo Scientific, Catalog # 89839). Tissue and cells were washed in PBS prior to Dounce homogenization on ice as follows: Liver 200 mg tissue, 70 strokes (loose); Spleen 50 strokes (tight); HEK293^Blue-hTLR9^ cells 50 strokes (tight). Density gradients were generated according to the manufacturer’s instructions and used to fractionate lysates by ultracentrifugation at 145,000 × *g* for 2 h at 4 °C. The lysosomal fraction was washed with PBS before the isolated pellet was frozen at −80 °C. Lysosomal-enriched nucleic acid was released from lysosomes after four freeze-thaw cycles between −80 ^o^C and 37 °C of lysosomal pellets in the presence of 1× polynucleotide kinase buffer. After insoluble debris was centrifuged away, the supernatant was radiolabeled with [γ-^32^P] ATP (Perkin Elmer) using T4 polynucleotide kinase (New England Biolabs) for 30 min at 37 °C. Samples were then denatured in 95% formamide and 10 mM EDTA at 70 °C for 5 min and fractionated on 20% acrylamide and urea gels in 50 mM histidine buffer^66^. Radiolabelled nucleic acids were detected using Typhoon 9410 Imager (Amersham Biosciences).

### Protein purification and nuclease assay

Recombinant, soluble PLD3, PLD3-AA, PLD4, and PLD4-AA proteins were prepared as described with minor modification^30^. Briefly, the plasmids encoding these proteins were transiently transfected into HEK293S GnTI cells at a density of 1 × 10^6^ cells per ml with PEI 40 K (Polysciences, 24765-1) according to manufacturer’s instructions. After transfection, the cells were incubated in a humidified 37 °C CO_2_ incubator (8%), rotating at 135 r.p.m. Cells were harvested 7 days post transfection, centrifuged at 3000 × *g* for 30 min, and the supernatant was filtered using a 0.2 μm filter (Millipore). Protino Ni-NTA Agarose beads (Macherey-Nagel, 745400.100) were added to the supernatants, followed by rotation for 3 h at 4 °C. After incubation, the beads were washed three times with washing buffer (20 mM Tris-HCl, pH 7.5, 250 mM NaCl and 10 mM imidazole) and eluted with elution buffer (20 mM Tris-HCl, pH 7.5, 250 mM NaCl and 200 mM imidazole). Buffer exchange of the eluted proteins to 1× PBS was performed with Amicon Ultra 15 ml filter with 30 kDa cut-off (Millipore Sigma, NUFC903024). Proteins were analyzed for purity by SDS-PAGE stained with Coomassie Brilliant Blue. Nuclease assay was essentially identical to the protocol described^30^ except that the substrate sequences were synthetic RNA oligonucleotides (IDT). Assays for digestion of RNA were carried out for 2 h at 37 °C in 50 mM 2-(N-morpholino) ethanesulfonic acid (MES), 125 mM NaCl, with substrates at 2.0 μM and PLD3 at 20 nM or PLD4 at 200 nM in the presence of 1 U/μl recombinant ribonuclease Inhibitor RNaseOUT (Thermo, 10777019). pH was 5.5 for PLD3 or 5.0 for PLD4, and in some experiments a pH range of 4.5–7.0 to assess optimal pH. After digestion, reaction mixtures were subjected to 10% or 15% denaturing Tris-Borate-EDTA PAGE (Thermo, EC68755BOX or EC68855BOX) and stained with SYBR gold (Thermo, S-11494). In Figs. 3 and 4a, b, reactions were performed in 50 mM Acetate 20 mM NaCl at either pH 5.2 (PLD3) or pH4.4 (PLD4).

### Anti-TLR9 treatment

The anti-mouse TLR9 antibody NaR9 has been described^67^. *Unc93b1*^+/3d^*Pld3*^−/−^*Pld4*^−/−^ mice, generated from (*Unc93b1*^3d/3d^*Pld3*^−/−^*Pld4*^−/−^ X *Pld3*^−/−^*Pld4*^+/–^) F1 breeding, were treated by injecting 100 μg antibody/ mouse in saline every 3 days starting at days 12–15. Control litters received IgG2a MAb with an irrelevant specificity. Healthy littermates (*Unc93b1*^+/3d^*Pld3*^−/−^*Pld4*^+/–^) were weaned day 17 and treated mice were kept on moist food after weaning on day 26 for the remainder of the experiment.

### Cell trace violet cell transfer

Pooled cells from spleen and lymph nodes from C57BL/6 and *Ifnar1*^*−/−*^ mice were labelled with 5 or 1 μM cell trace violet respectively (according to manufacturer’s instructions). After washing in saline, 4 × 10^6^ cells were injected intravenously into animals of each genotype. Four days later, lymph nodes were harvested and pooled, and cell trace violet-positive cells were assessed for expression of Ly6C and cell-specific markers.

### Reporting summary

Further information on research design is available in the Nature Research Reporting Summary linked to this article.

## Supplementary information

Supplementary Information

Description of Additional Supplementary Files

Supplementary Data 1

Reporting Summary

## Data Availability

All data are available upon request to the corresponding author. Raw data including original gel scans from Figs. 1a, c–h, 2b–h, 3b–g, 4a–f, 5a–c, 6a–c, 7b, d, 8b–j and Supplementary Figs. 1, 3–12 are given in the accompanying source data. Read length data from Fig. 5a, b is also provided in Supplementary Tables 3 and 4. Processed RNA seq data of Supplementary Fig. 14 is provided in Supplementary Data 1. The original RNAseq data have been deposited in the GEO database, and are available under the primary accession code GSE182648. Materials such as unique cell lines or mice are available upon execution of material transfer agreement with The Scripps Research Institute by request from the corresponding author. Source data are provided with this paper.

## Source data

Source Data

## Code availability

No new code was generated for these studies.
