# Supplementary material for: Cleavage of DNA and RNA by PLD3 and PLD4 limits autoinflammatory triggering by multiple sensors
Source: Nat Commun. 2021 Oct 7;12:5874. doi: 10.1038/s41467-021-26150-w (PMC8497607; doi:10.1038/s41467-021-26150-w)
Supplement: Supplementary file 1 — Supplementary Information [file 41467_2021_26150_MOESM1_ESM.pdf]

Supplementary Tables and Figures

Cleavage of DNA and RNA by PLD3 and PLD4 limits autoinflammation triggered by multiple sensors

Amanda L. Gavin[1], Deli Huang[1], Tanya R. Blane[1], Therese C. Thinnes[1], Yusuke Murakami[2], Ryutaro Fukui[3], Kensuke Miyake[3], David Nemazee[1]*

This file contains 15 Supplementary Figures with accompanying legends followed by 4 Supplementary Tables.

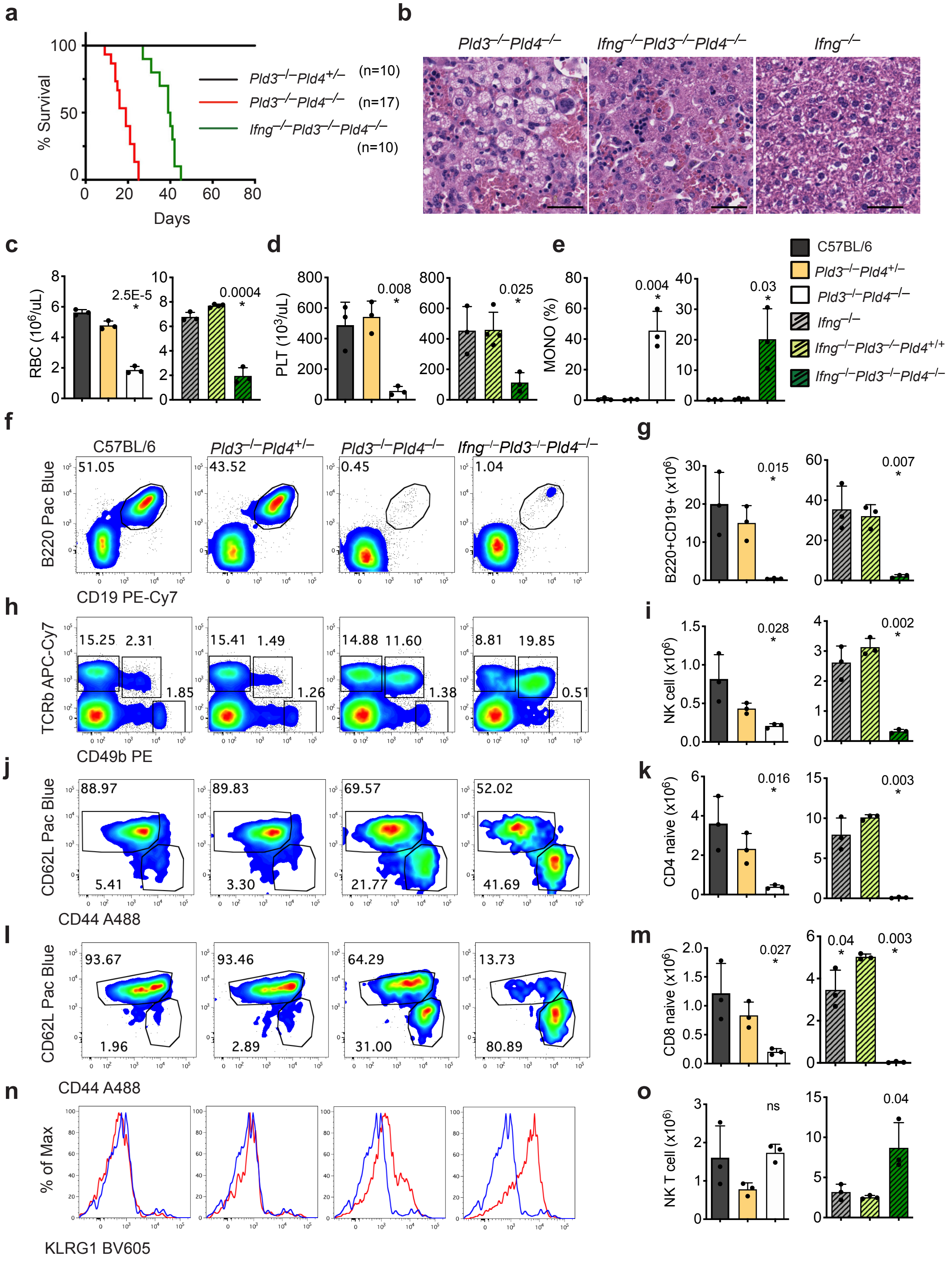

Supplementary Figure 1. Analysis of the role of IFN-γ on survival and immunopathology of *Pld3*$^{-/-}$*Pld4*$^{-/-}$ mice.

Comparison of mice deficient in one or more genes encoding PLD3, PLD4, or IFN-γ. **a** Analysis of survival of *Pld3*$^{-/-}$*Pld4*$^{-/-}$ compared to *Ifng*$^{-/-}$*Pld3*$^{-/-}$*Pld4*$^{-/-}$ and *Pld3*$^{-/-}$*Pld4*$^{+/-}$ mice: median survival in days was 19, 39.5, >150, respectively, with group size n= 17, 10, 10, respectively. Survival was reduced significantly in *Pld3*$^{-/-}$*Pld4*$^{-/-}$ and *Ifng*$^{-/-}$*Pld3*$^{-/-}$*Pld4*$^{-/-}$ groups compared to *Pld3*$^{-/-}$*Pld4*$^{+/-}$ mice (Chi squared, 23.98, 21.32, respectively, by log rank test, with p<.0001 for both comparisons.) **b** Comparison of liver histopathology of the indicated strains by H&E stain. Scale bar is 40 μm. **c-e** Analysis of frequencies of selected blood cells: **c)** erythrocytes (RBC); **d** platelets (PLT); **e** monocytes (Mono). **f-o** Flow cytometry analysis of lymphocyte subsets in the spleen. Mice of the indicated genotypes were assessed for the numbers of the following cell types. **f,g** B220$^+$CD19$^+$ B cells; **h,i** CD49b$^+$TCRb$^-$ NK cells; **j,k** CD62L$^+$CD44^{lo} naive CD4$^+$ T cells; **l,m** CD62L$^+$CD44^{lo} naive CD8$^+$ T cells; and **n,o** CD49b$^+$TCRb$^+$ NK$^-$T cells. **n)** KLRG1 levels on gated NK-T cells from h. Bar graphs show mean ± SD. Unpaired two-tailed T-test statistical comparisons in g,i,k,m,o were between *Pld3*$^{-/-}$*Pld4*$^{-/-}$ and C57BL/6 groups (left panels, mouse ages were 15-16 days-old) or between *Ifng*$^{-/-}$*Pld3*$^{-/-}$*Pld4*$^{-/-}$ and *Ifng*$^{-/-}$ groups (right panels, mouse ages were 26-30 days-old). In plots c,d,e,g,I,k,m and o, each bar represents mean, error bars show SD, and each symbol represents value obtained in an individual mouse (n=3/ group). These experiments were performed twice with similar results.

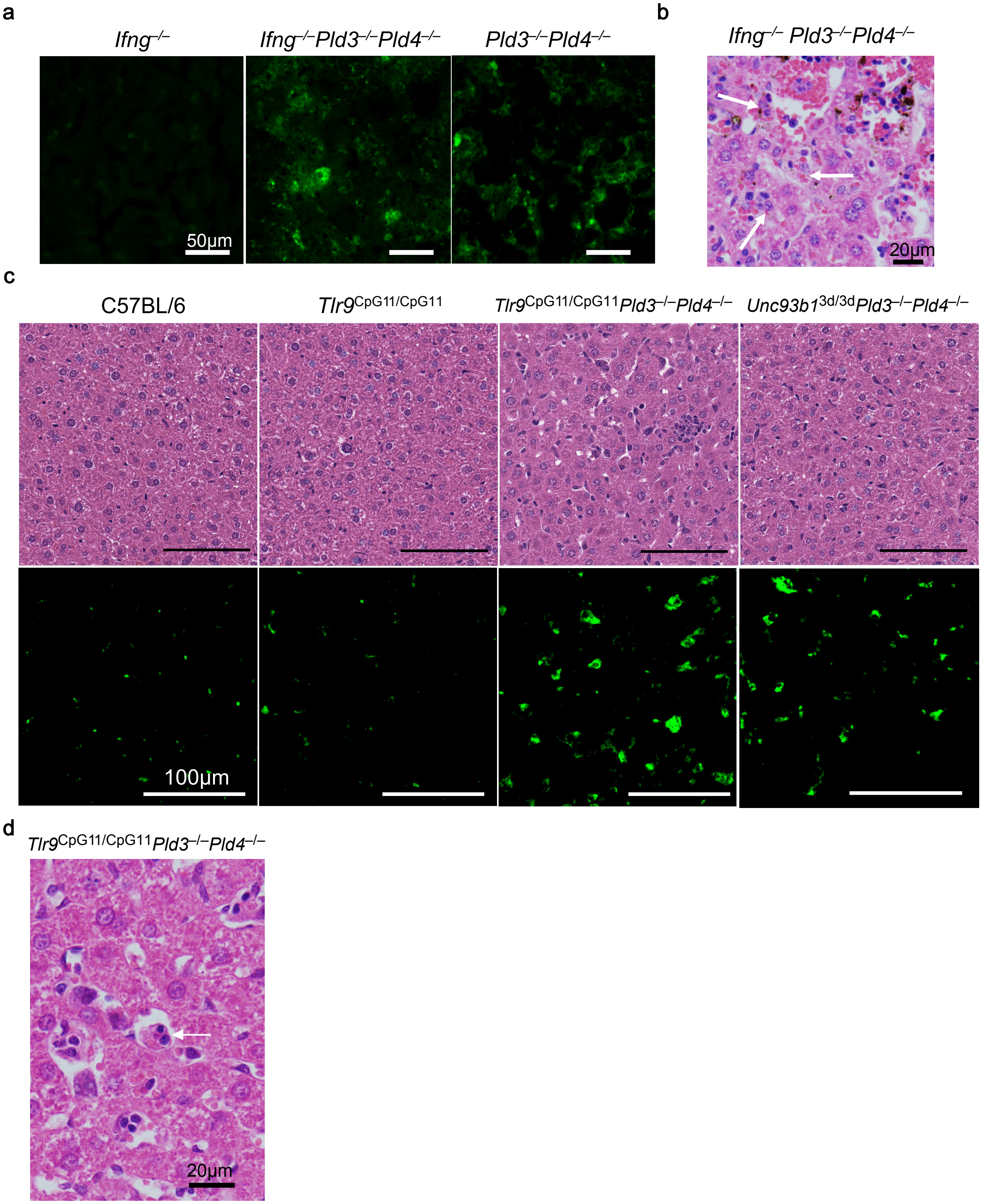

Supplementary Figure 2. Liver phenotypes in *Pld3*$^{-/-}$*Pld4*$^{-/-}$ mice and the effects of additional mutations.

CD68 frozen section stains or hematoxylin & eosin stains of livers of mice of the indicated genotypes. **a** Shows elevated CD68 staining in *Pld3*$^{-/-}$*Pld4*$^{-/-}$ and *Ifng*$^{-/-}$*Pld3*$^{-/-}$*Pld4*$^{-/-}$ samples compared to *Ifng*$^{-/-}$ control. Scale bar indicates 50 μm. **b** Higher power magnification showing hemophagocytosis in *Ifng*$^{-/-}$*Pld3*$^{-/-}$*Pld4*$^{-/-}$ liver (indicated by arrows). Scale bar indicates 20 μm. **c** Diminished pathology revealed by H&E stain (upper panels) but continued CD68^{+} myeloid cell influx in *Unc93b1*$^{3d/3d}$*Pld3*$^{-/-}$*Pld4*$^{-/-}$ livers (lower panels). Scale bar indicates 100 μm. **d** Hemophagocytosis in *Tlr9*$^{CpG11/Cpg11}$*Pld3*$^{-/-}$*Pld4*$^{-/-}$ liver sample (arrow). Scale bar indicates 20 μm. Liver lobes from four animals of each genotype were sectioned for histological analysis.

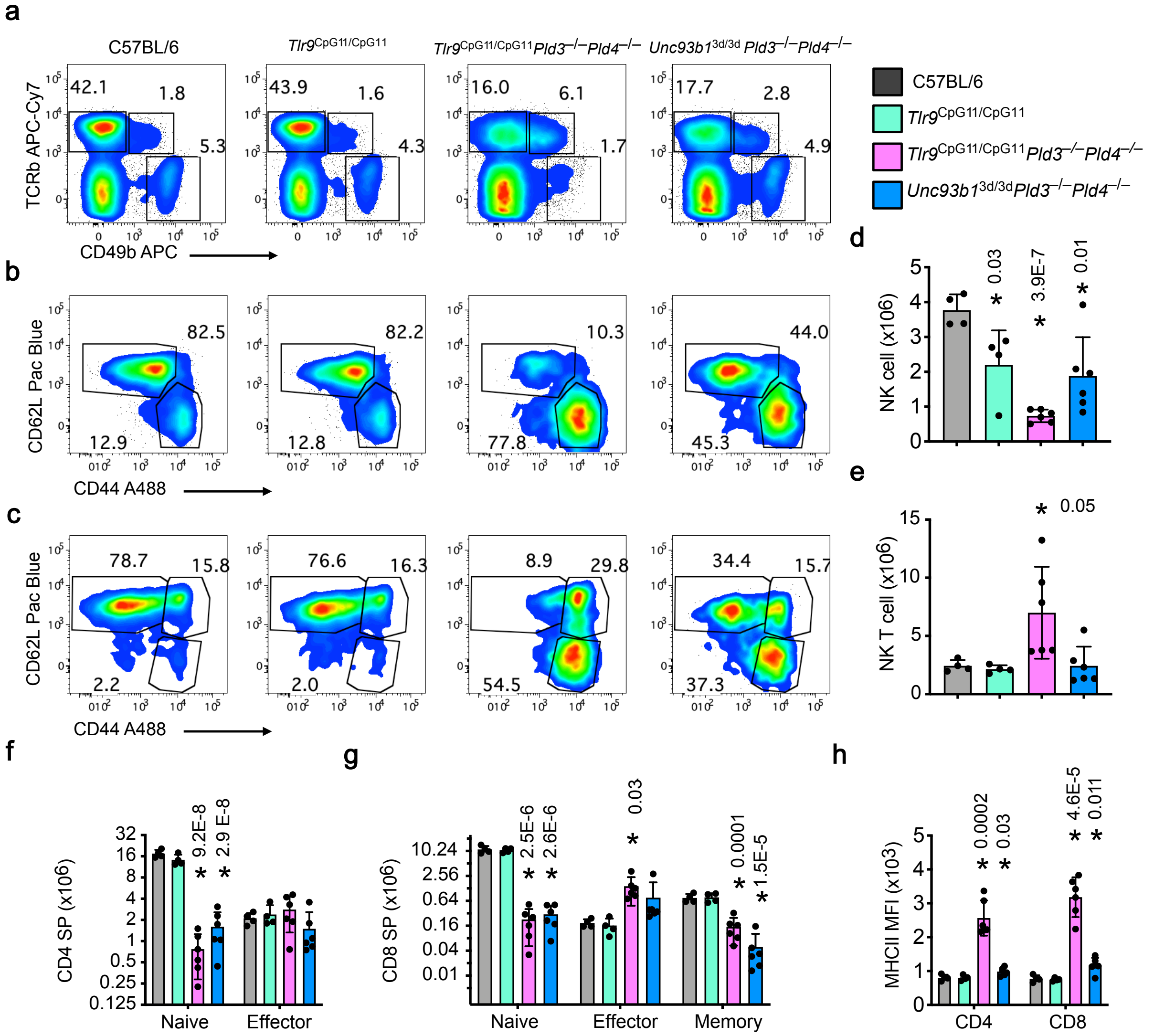

Supplementary Figure 3. T cell and NK cell abnormalities in *Tlr9*$^{CpG11/CpG11}$*Pld3*$^{-/-}$*Pld4*$^{-/-}$ mice. Flow cytometry analysis of splenic populations. **a** Shows flow cytometry gating strategy for T cells, NK-T and NK populations. Plots in **b,c** show CD62L and CD44 expression of CD4^{+} and CD8^{+} T cells, respectively. **d-g** Plots show absolute numbers of cells of the indicated cell types in the spleens of mice of the indicated genotypes. **d** NK, **e** NK-T, **f** Naive and effector CD4^{+} T, **g** Naive, effector and memory CD8^{+} T. **h** Analysis of MHCII levels on naive CD4^{+} and CD8^{+} T cells. Asterisks below p-values (Two-tailed, unpaired T-test) compare strains indicated to C57BL/6 splenocyte subsets. Bar graphs show mean ± SD with each symbol representing an individual mouse. This experiment was performed at least twice with similar results.

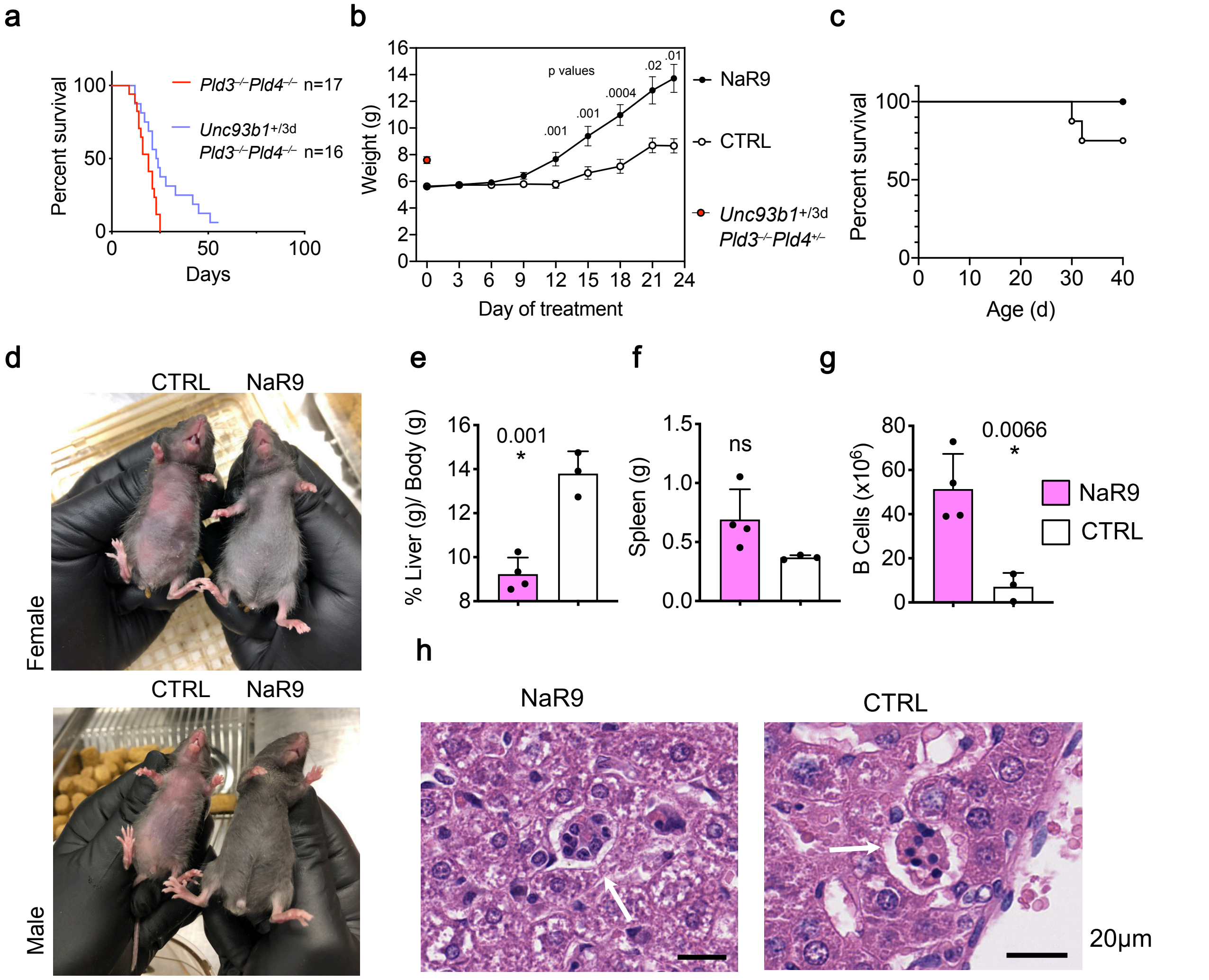

Supplementary Figure 4. Therapeutic response of *Unc93b1*$^{+/3d}$*Pld3*$^{-/-}$*Pld4*$^{-/-}$ mice to treatment with anti-TLR9. **a** Comparison of survival of untreated *Unc93b1*$^{+/3d}$*Pld3*$^{-/-}$*Pld4*$^{-/-}$ mice compared to *Pld3*$^{-/-}$*Pld4*$^{-/-}$ mice. Median survival was 23.5 d for *Unc93b1*$^{+/3d}$*Pld3*$^{-/-}$*Pld4*$^{-/-}$ mice compared to 19 d for *Pld3*$^{-/-}$*Pld4*$^{-/-}$ mice. p=.0087 rank log test. **b-h** Cohorts of *Unc93b1*$^{+/3d}$*Pld3*$^{-/-}$*Pld4*$^{-/-}$ mice were either treated every third day with 100 µg/mouse anti-TLR9 MAb NaR9 or isotype control starting at day 12 after birth and monitored for the following parameters. **b** Body weight: Control, *Unc93b1*$^{+/3d}$*Pld3*$^{-/-}$*Pld4*$^{+/-}$ littermates (red symbol) were weighed at the start of the experiment. NaR9-treated (n=4) or control Ig-treated (n=4) *Unc93b1*$^{+/3d}$*Pld3*$^{-/-}$*Pld4*$^{-/-}$ mice were weighed on the indicated days. **c** Combined survival from two pooled experiments (n=8 NaR9 and n=8 control). **d** Appearance of mice (females upper panel, males lower panel) at day 18 of antibody treatment indicated. **e** Liver as

a percentage of body mass, **f** Spleen weight, **g** Splenic B cell numbers, **h** Analysis of liver histology with arrows indicating hemophagocytosis. Bar graphs show mean ± SD with each symbol representing an individual mouse. Statistical analysis in b, e-g was two-tailed T test. This experiment was performed twice with similar results.

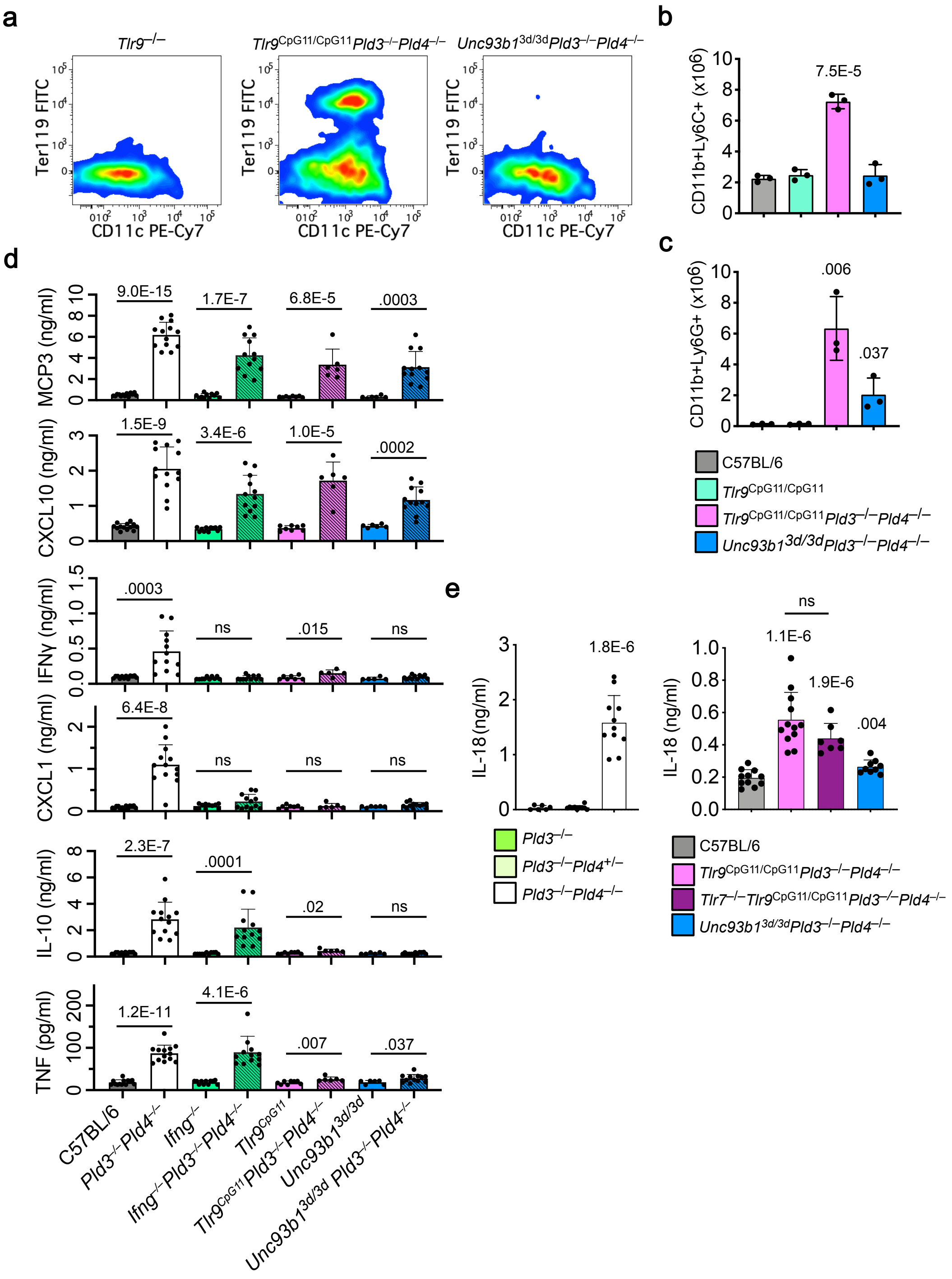

Supplementary Figure 5. Ameliorated disease in *Unc93b1*$^{3d/3d}$*Pld3*$^{-/-}$*Pld4*$^{-/-}$ compared to *Tlr9*$^{CpG11/CpG11}$*Pld3*$^{-/-}$*Pld4*$^{-/-}$ mice.

The indicated mouse strains were compared by flow cytometry for altered cell phenotypes and analyzed for alterations in serum cytokines and chemokines. **a** Flow cytometry analysis of hemophagocytosis indicated by Ter119^{+}CD11c^{+}CD11b^{+} cells in spleen. **b** Analysis of splenic myeloid

cell numbers. **c** Analysis of elevated spleen neutrophil number. **d** Serum cytokine analysis. Sera from mice were taken at 2 wk for all *Pld3*$^{-/-}$*Pld4*$^{-/-}$ and *Ifng*$^{-/-}$*Pld3*$^{-/-}$*Pld4*$^{-/-}$ mice and for an equal number of their contemporaneous wild-type controls. Sera from all other mice were taken at 2-3 months of age. Unpaired two-tailed T-test was performed comparing groups depicted with line. **e** Concentration of IL-18 in sera from mice of the indicated genotypes and ages. Littermate animals that were 16 days old are shown on left, while non littermate animals that were 6-10 weeks old are compared on the right. Bar graphs show mean ± SD, with symbols depicting individual mice. Experiments shown in a-c were performed at least twice with similar results. In b,c values are from 3 independent mice/group. In d, serum samples were from the following number of independent mice: C57BL/6, n=13; *Pld3*$^{-/-}$*Pld4*$^{-/-}$, n=13; *Ifng*$^{-/-}$, n=11; *Ifng*$^{-/-}$*Pld3*$^{-/-}$*Pld4*$^{-/-}$, n=12; *Tlr9*$^{CpG11/CpG11}$, n=8; *Tlr9*$^{CpG11/CpG11}$*Pld3*$^{-/-}$*Pld4*$^{-/-}$, n=6; *Unc93b1*$^{3d/3d}$, n=6; *Unc93b1*$^{3d/3d}$*Pld3*$^{-/-}$*Pld4*$^{-/-}$, n=12. In e, serum samples were from independent mice: *Pld3*$^{-/-}$, n=6; *Pld3*$^{-/-}$*Pld4*$^{+/-}$, n=10; *Pld3*$^{-/-}$*Pld4*$^{-/-}$, n=11; C57BL/6, n=11; *Tlr9*$^{CpG11/CpG11}$*Pld3*$^{-/-}$*Pld4*$^{-/-}$, n=12; *Tlr7*$^{-/-}$*Tlr9*$^{CpG11/CpG11}$*Pld3*$^{-/-}$*Pld4*$^{-/-}$, n=7; *Unc93b1*$^{3d/3d}$*Pld3*$^{-/-}$*Pld4*$^{-/-}$, n=9;

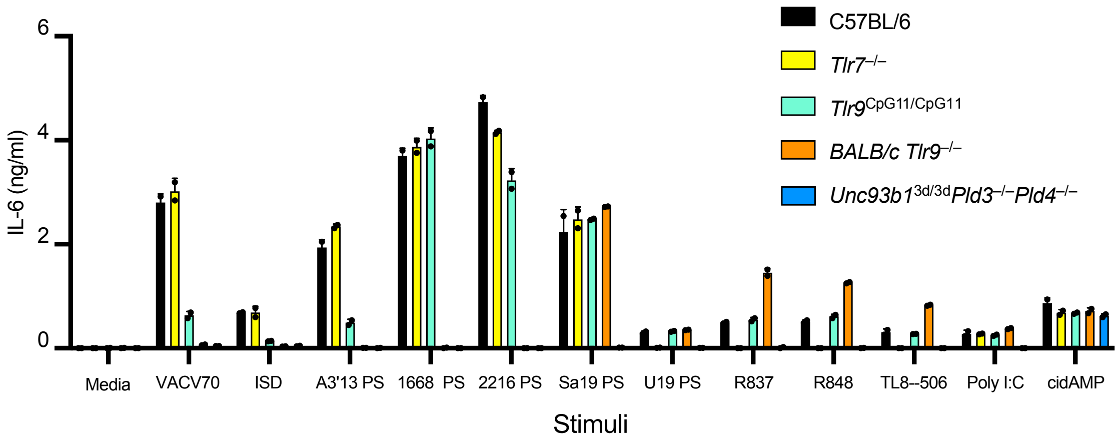

Supplementary Figure 6. Effect of *Tlr9*$^{CpG11/CpG11}$ (missense) or *Tlr9*$^{-/-}$ (null) mutations on IL-6 responses of GM-CSF-elicited bone marrow DCs to a variety of stimuli of nucleic acid sensors. Note the residual (and to phosphorothioate ODNs sometimes substantial) responses of *Tlr9*$^{CpG11/CpG11}$ but not *Tlr9*$^{-/-}$ DCs to known oligodeoxynucleotide ligands of TLR9 and the augmentation of some TLR7 responses in *Tlr9*$^{-/-}$ but not *Tlr9*$^{CpG11/CpG11}$ DCs. Responses to the TLR13 ligand Sa19-PS were similar in all UNC93b1-sufficient DCs tested.

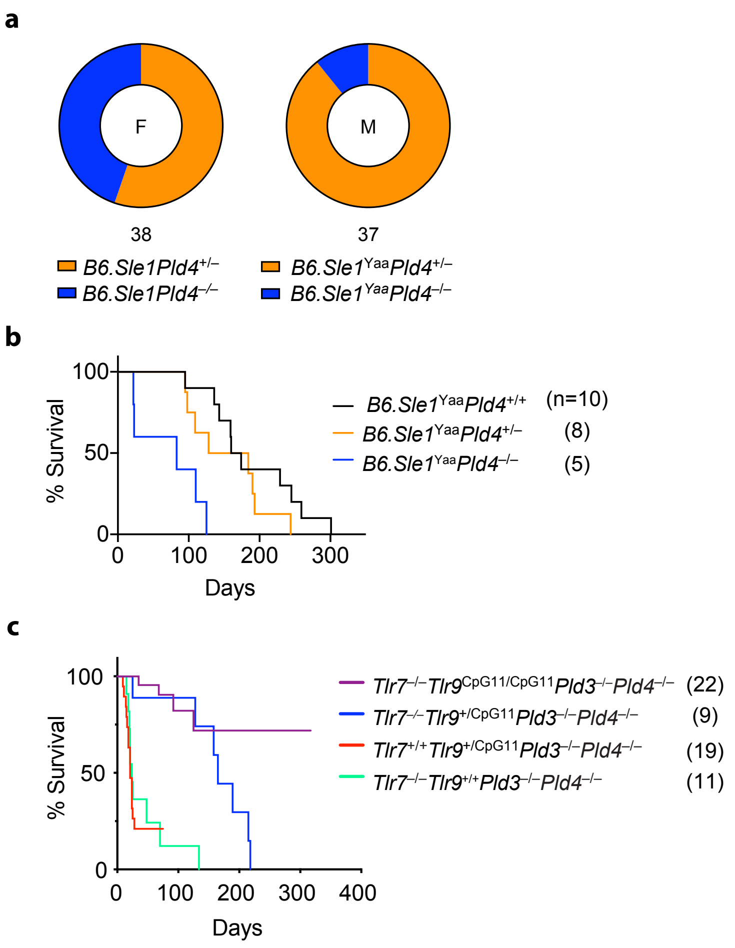

Supplementary Figure 7. TLR7 signaling promotes reduced survival in mice lacking PLD4 or both PLD3 and PLD4.

a,b Analysis of lupus-prone $B6.Sle1Y^{aa}$ mice lacking PLD4. A total of 38 female and 37 male offspring of ($B6.Sle1Pld4^{-/-}$ x $B6.Sle1Y^{aa}Pld4^{+/-}$) breeding were genotyped. **a** Reduced recovery of PLD4-deficient male mice at weaning. Expected proportions are 50% for the ($B6.Sle1Pld4^{-/-}$ x $B6.Sle1Y^{aa}Pld4^{+/-}$) breeding (p= .51 for females, p= 0.00000186 for males, χ^2 test). **b** Survival of the male $B6.Sle1Y^{aa}$ cohorts deficient in 0, 1, or 2 functional *Pld4* genes. **c** Rescue in survival of $Pld3^{-/-}Pld4^{-/-}$ mice carrying mutations in *Tlr9*, *Tlr7* or both. Note that $Tlr7^{-/-}Tlr9^{+/CpG11}Pld3^{-/-}Pld4^{-/-}$ mice had delayed death (185 d) compared to $Tlr7^{+/+}Tlr9^{+/CpG11}Pld3^{-/-}Pld4^{-/-}$ mice (21 d), p=0.0012 log rank test.

The numbers of independent mice for each group are indicated.

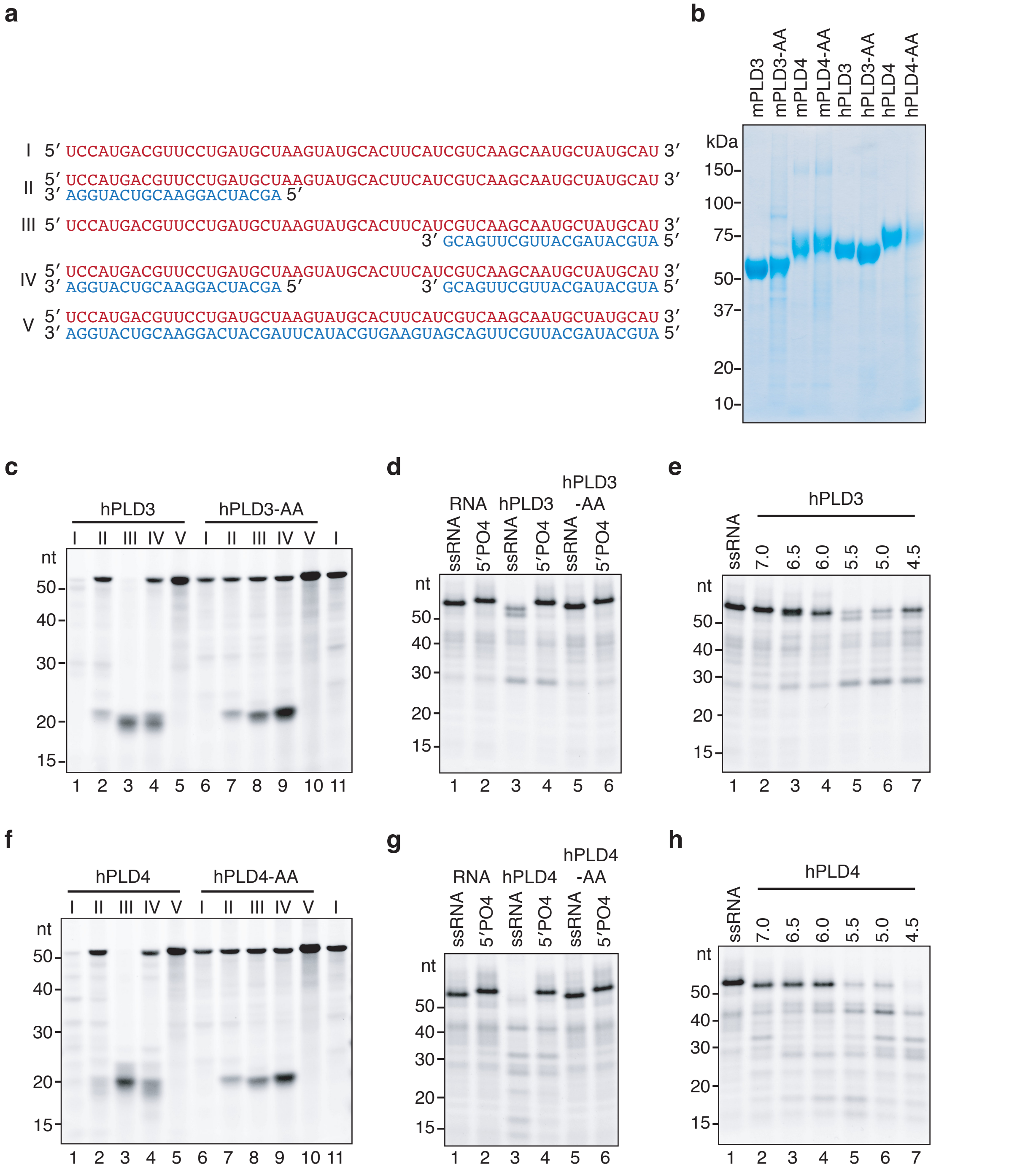

Supplementary Figure 8. RNAse activity of human PLD3 and human PLD4.

These experiments were carried out identically to those of Figure 3 but with recombinant soluble PLD3 and PLD4 of human rather than mouse sequence. **a** Nucleotide sequences and schematic structures of substrates (I-V) used. Note that the red strand is identical to "I" in all substrates and the blue strands are partly or completely complementary, annealed strands. **b** Coomassie blue staining of denaturing protein gel showing purity of isolated recombinant soluble PLD3, PLD3-AA, PLD4 and

PLD4-AA from mouse and human used in this study. **c** Denaturing 10% Tris-Borate-EDTA PAGE and SYBR gold visualization of the products of incubation of PLD3 or enzymatically dead mutant PLD3-AA with the RNA substrates I-V, as indicated above the lanes. **d** Products of substrate I with or without phosphorylation at the 5′ end (5′PO_4) digested by human PLD3 or PLD3-AA as in **c** except a 15% gel was used. **e** PAGE analysis of products of incubation of substrate I as in **c**, but with buffers adjusted to a pH of 4.5-7.0 and use of a 15% gel. **f-h** Digestion of RNA by PLD4 or PLD4-AA control enzyme. Conditions and substrates were as indicated in c,e except that enzymes were used at 200 nM and in f,g the pH was 5.0. These experiments were repeated once.

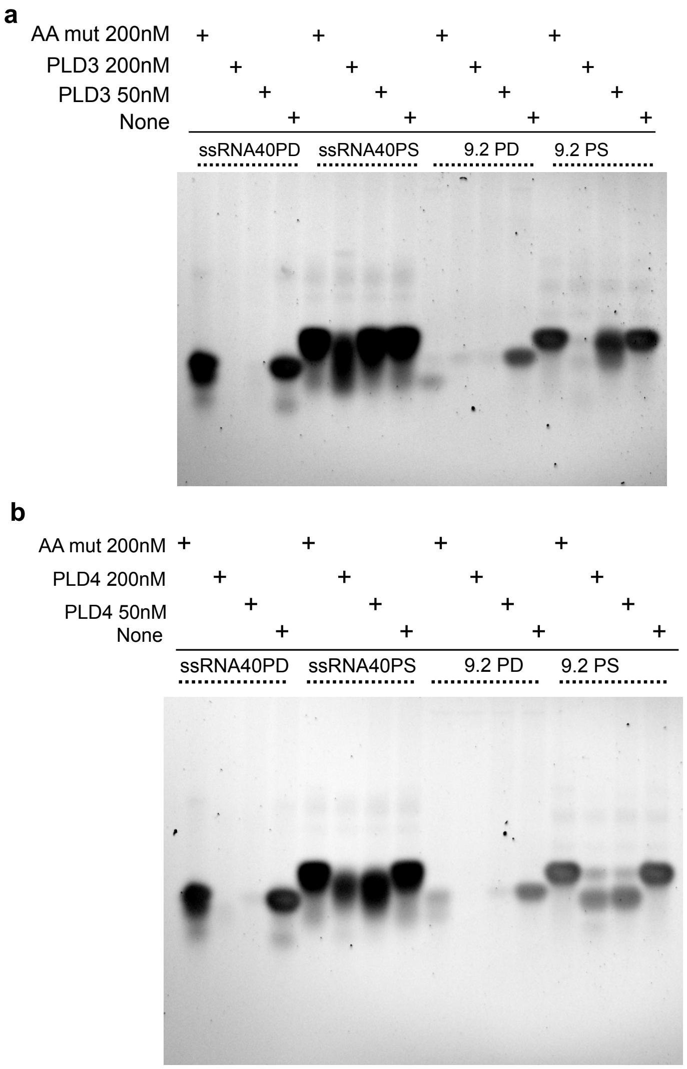

Supplementary Figure 9. In vitro digestion of oligoribonucleotide TLR7 ligands by PLD3 and PLD4.

Phosphodiester and phosphorothioate forms of ssRNAs 9.2 and ssRNA40 were digested with the indicated amounts of wild type or inactive **a** PLD3 and **b** PLD4. Each substrate was at 2 μM. Reactions were performed for 6 hours at 37°C in 50 mM Acetate 20 mM NaCl at pH 5.2 for PLD3 or at pH 4.4 for PLD4. None indicates no addition of PLD3 and PLD4 for the digestion period.

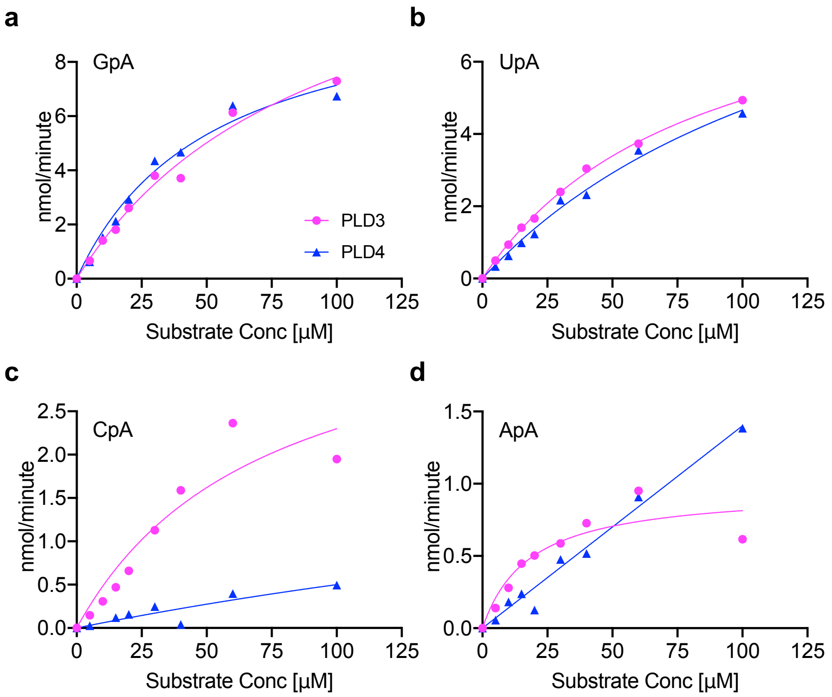

Supplementary Figure 10. Michaelis–Menten curves of diribonucleotide digestion by human recombinant soluble PLD3 and PLD4.

The indicated dinucleotides at a range of concentrations from 0 to 100 μM were incubated with 25 nM PLD3 (pink) or PLD4 (blue) along with 2 μg/ml adenosine deaminase and digestion was followed spectroscopically as in Fig 4.

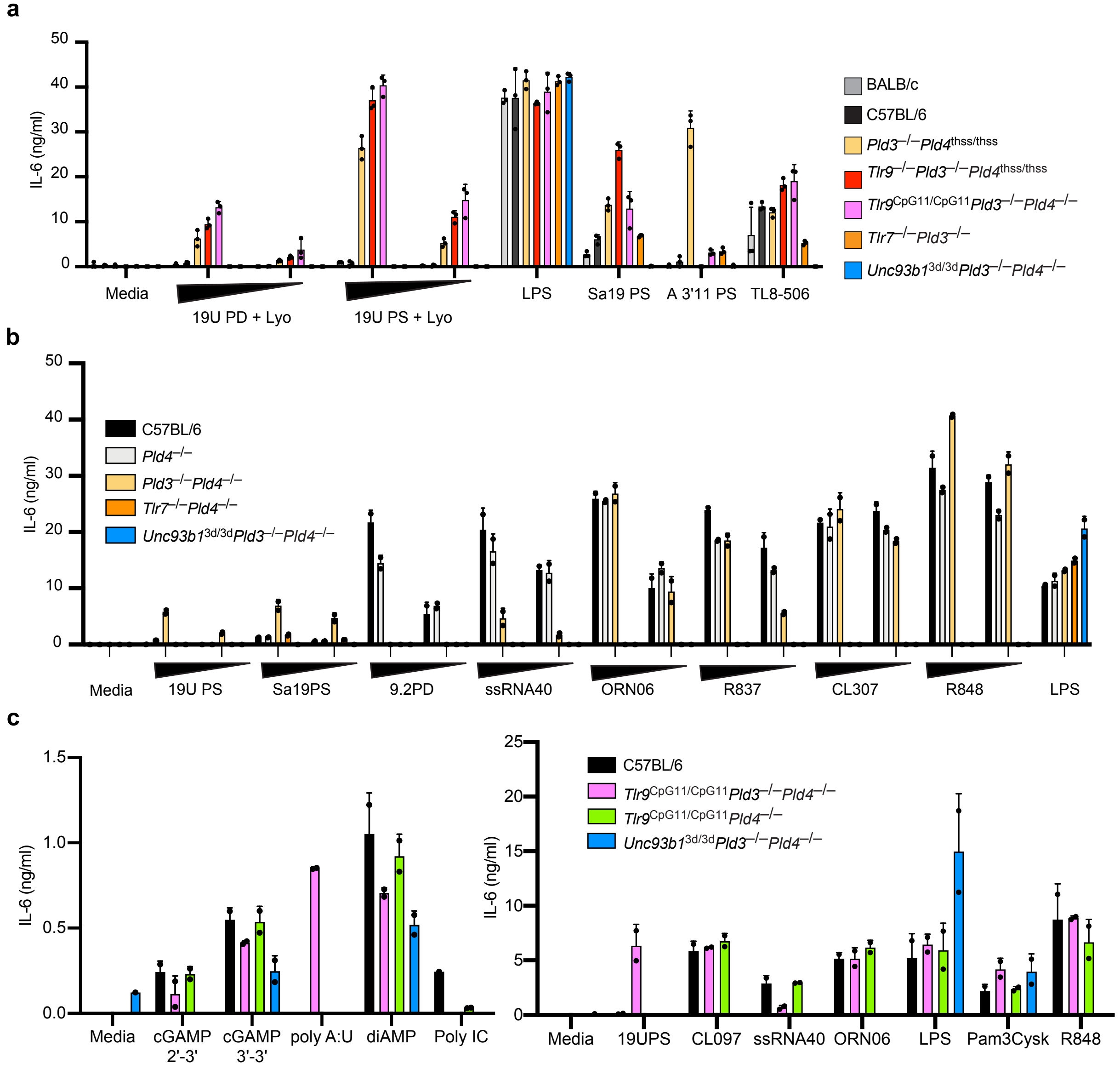

Supplementary Figure 11. Analysis of IL-6 responses to a variety of ligands by GM-CSF-elicited DCs lacking PLD3 and PLD4.

Cells of the indicated genotypes were challenged with the following ligands for TLR7 (U$_{19}$, 9.2-PD, ssRNA40, R837, CL307, R848), mTLR8 (TL8-506), TLR4 (LPS), STING (cGAMP 2′-3′, cGAMP 3′-3′, c-diAMP), TLR3 (poly A:U, poly I:C), TLR1/2 (Pam3CSK4). **a** Note the exaggerated IL-6 response by *Pld3*$^{-/-}$*Pld4*$^{-/-}$ cells to either phosphodiester (PD) poly U (19U) or phosphorothioate-stabilized (PS) 19U over a six-fold dose range (depicted by black triangle) irrespective of no (*Tlr9*$^{-/-}$*Pld3*$^{-/-}$*Pld4*$^{thss/thss}$), partial (*Tlr9*$^{Cpg11/Cpg11}$*Pld3*$^{-/-}$*Pld4*$^{-/-}$), or wild type (*Pld3*$^{-/-}$*Pld4*$^{-/-}$) levels of TLR9 function. **b** Analysis of responses to TLR7 and TLR13 ligands at various doses in TLR9-sufficient cells carrying the indicated

mutations. Note that in the absence of both PLD3 and PLD4 some responses are enhanced (19U and Sa19), equivalent (ORN06, R848 and CL307) or reduced (ssRNA40, 9.2 PD) compared to control C57BL/6 cell responses. **c** Comparative IL-6 responses to activators of STING, TLR4, TLR2/1, TLR3 and TLR7 pathways.

a

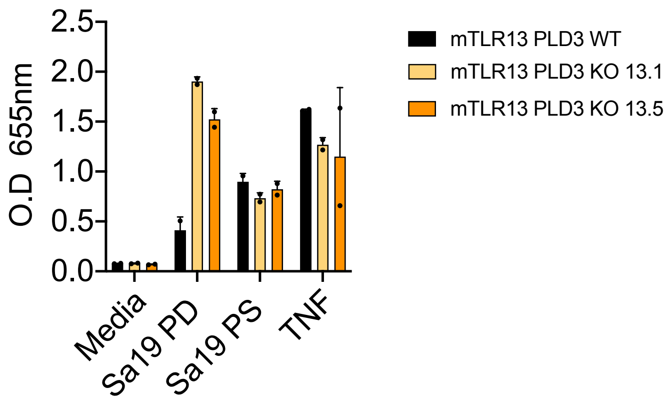

b

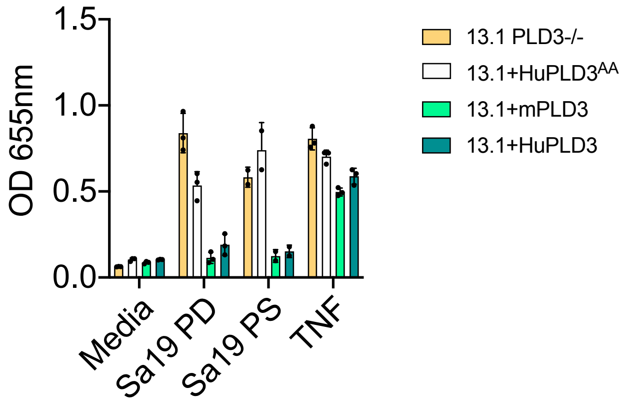

Supplementary Figure 12. Analysis of NF-κB reporter responses of PLD3-deficient or PLD3-sufficient HEK-Blue^{mTLR13} cells elicited by TLR13 ligands ORNSa19 and ORNSa19PS, or TNF positive control.

a The effect of PLD3/4 deficiency on responses to the TLR13 ligand Sa19 (5'-GGACGGAAAGACCCCGUGG-3') or its phosphorothioate-linked derivative Sa19-PS in HEK-Blue™-mTLR13 reporter cells, which lack PLD4 expression, was tested by mutating *PLD3* using CRISPR. Two independent *PLD3*$^{-/-}$ clones were tested for responsiveness to Sa19 and Sa19-PS compared to unmutated cells. These *PLD3*$^{-/-}$ clones revealed a sensitization of the response to the nuclease sensitive ligand Sa19, while responses to Sa19-PS were similar in PLD3-deficient vs sufficient cells. **b** Clone 13.1 *PLD3*$^{-/-}$ cells were reconstituted with either wild type mouse (mPLD3) or human (HuPLD3) or HKD mutant PLD3 (HuPLD3^{AA}) and the effects on TLR13 responses was measured.

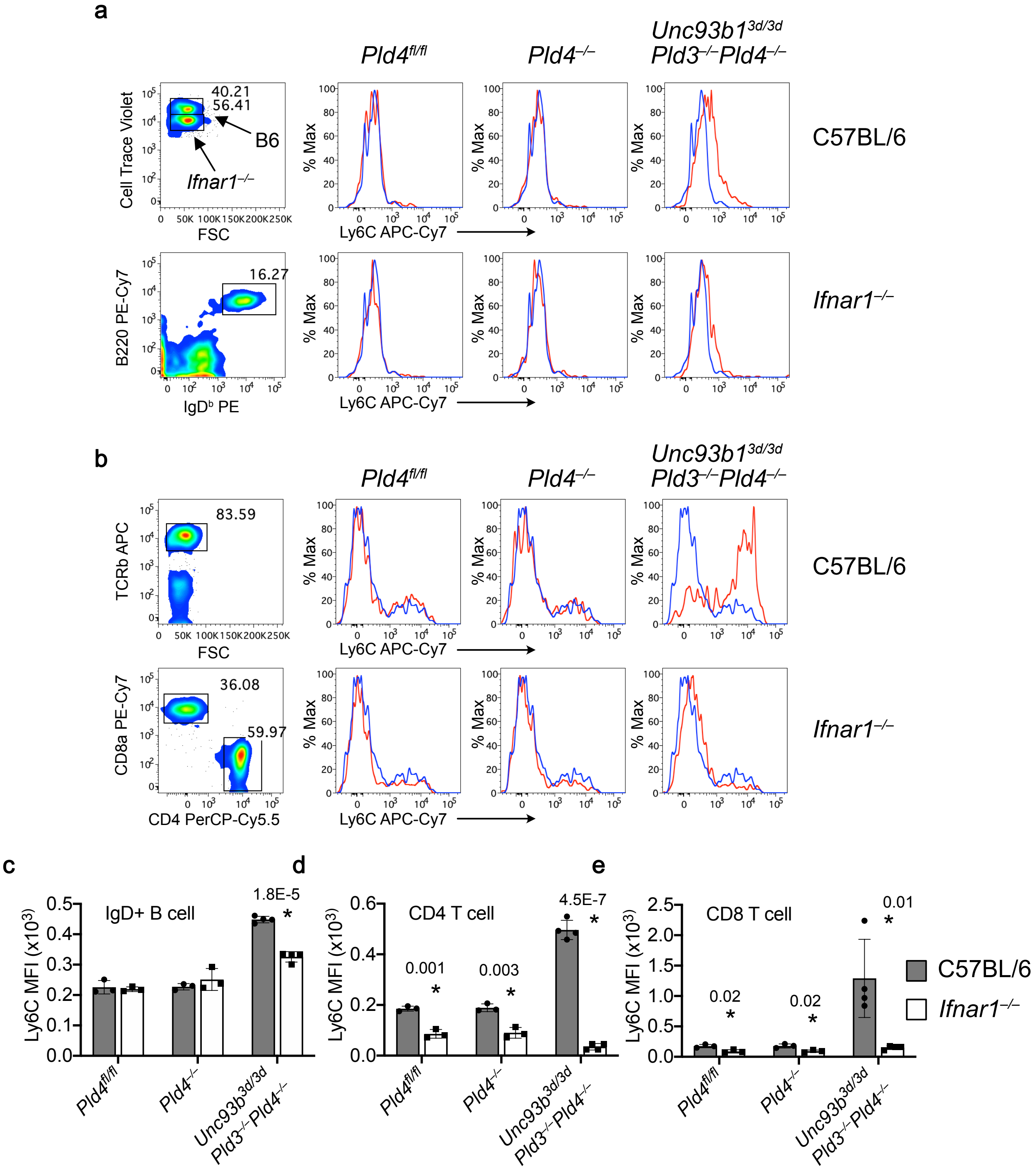

Supplementary Figure 13. Biological evidence for elevated type I IFN production in *Unc93b1*$^{3d/3d}$*Pld3*$^{-/-}$*Pld4*$^{-/-}$ mice.

Splenocytes of wild-type (B6) or *Ifnar1*$^{-/-}$ mice were labeled with different levels of cell trace violet, mixed and transferred i.v. to *Pld4*$^{fl/fl}$ (n=3), *Pld4*$^{-/-}$ (n=3), or *Unc93b1*$^{3d/3d}$*Pld3*$^{-/-}$*Pld4*$^{-/-}$ mice (n=4). After three days, pooled lymph node cells were recovered and assessed for upregulation of Ly6C. **a** Gating

on CTV$^+$ B cells reveals modest Ly6C upregulation only in *Unc93b1*$^{3d/3d}$*Pld3*$^{-/-}$*Pld4*$^{-/-}$ hosts and upregulation is lacking in *Ifnar1*$^{-/-}$ B cells. Blue traces show WT control mouse cells from an unmanipulated mouse compared to the indicated experimental transfer gated cells in red. **b** Analysis of transferred CD8$^+$ T cells in the same experiment with gating indicated on left, again showing significant upregulation of Ly6C only by WT cells in *Unc93b1*$^{3d/3d}$*Pld3*$^{-/-}$*Pld4*$^{-/-}$ hosts. **c-e** Quantitation of mean fluorescence intensity of Ly6C on the surface of the indicated cell subsets gated from this experiment. Bar graphs show mean ± SD, with symbols depicting individual recipient mice. Unpaired T-test compares Ly6C expression between C57BL/6 and *Ifnar1*$^{-/-}$ derived B cells from each recipient group. Experiment was performed once.

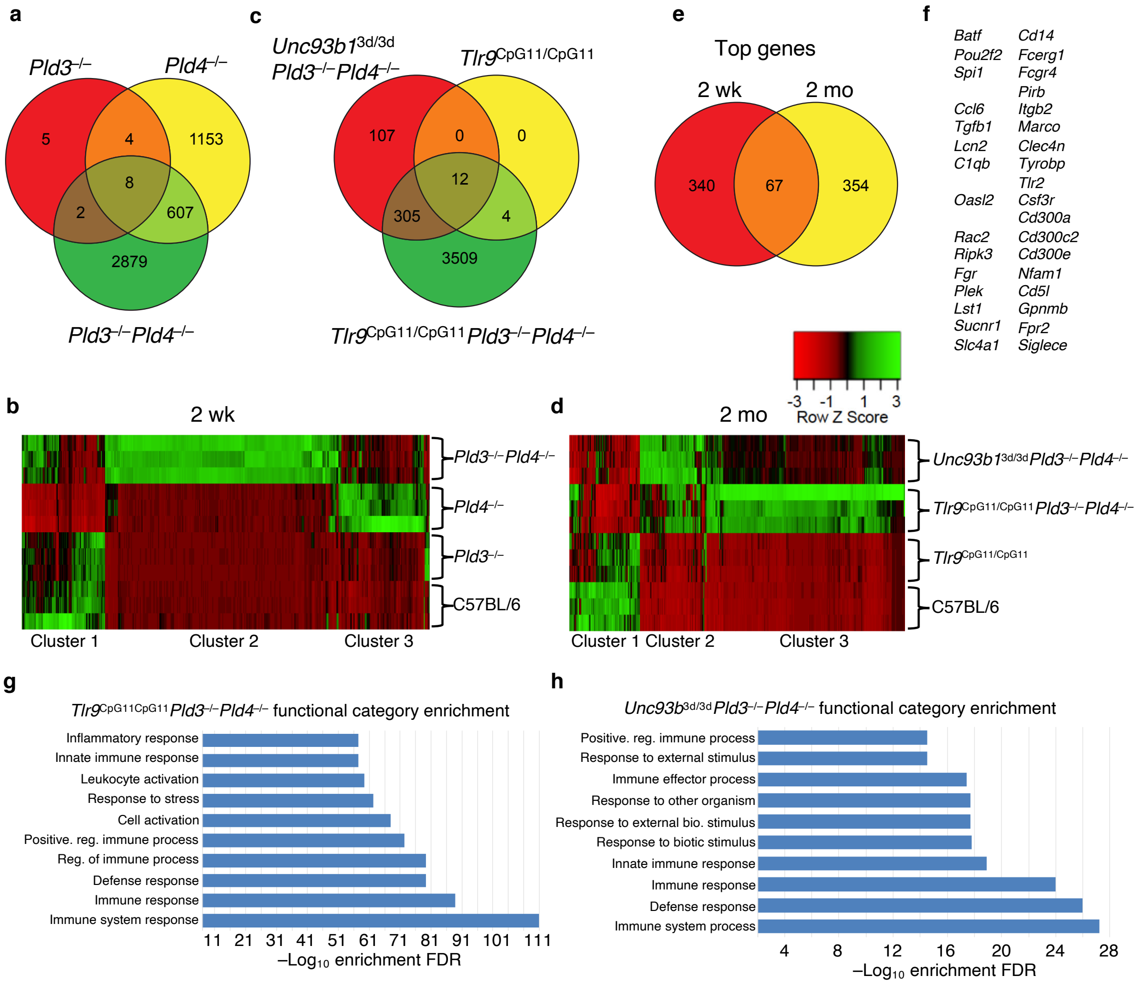

Supplementary Figure 14. RNAseq analysis of *Pld3*$^{-/-}$*Pld4*$^{-/-}$ liver.

Poly A+ RNA from whole livers were compared using next generation sequencing and the genes showing significant differences in expression from control livers of age-matched mice were further analyzed as follows. **a,c** Venn diagram analysis (https://www.bioinformatics.org/gvenn/) comparing the number of genes significantly different from C57BL/6 mice (FDR<.05) that were shared or distinct among the livers of the indicated genotypes (n=3/group). Mice ages were: **a** 2 weeks old, or **c** two months old. **b,d** Shown are differential expression plots generated by Heatmapper comparing relative RNA abundance in liver tissue of the indicated groups. A global view is shown highlighting Clusters 1-3 with distinct patterns of differential expression. **a,b** Comparison of liver RNAs from 2-wk-old C57BL/6, *Pld3*$^{-/-}$, *Pld4*$^{-/-}$, and *Pld3*$^{-/-}$*Pld4*$^{-/-}$ mice. n=3 mice/group. **c,d** Comparison of liver RNAs from 2-mo-old C57BL/6, *Tlr9*$^{CpG11/CpG11}$, *Tlr9*$^{CpG11/CpG11}$*Pld3*$^{-/-}$*Pld4*$^{-/-}$ and *Unc93b1*$^{3d/3d}$*Pld3*$^{-/-}$*Pld4*$^{-/-}$

mice. n=3 mice/group. Genes analyzed in b,d show those with the most statistically significant variations between groups and >2X difference in expression. **e,f** Shown is a comparison of the two experiments highlighting the most significant genes in terms of p-value and fold difference and their differences in the two experiments. **f** Of the 67 genes in common in e, annotated genes are shown. **g,h** Pathway analysis (http://bioinformatics.sdstate.edu/go/) showing the top ten functional categories with most significant gene enrichment in *Tlr9*$^{CpG11/CpG11}$*Pld3*$^{–/–}$*Pld4*$^{–/–}$ and *Unc93b1*$^{3d/3d}$*Pld3*$^{–/–}$*Pld4*$^{–/–}$ livers.

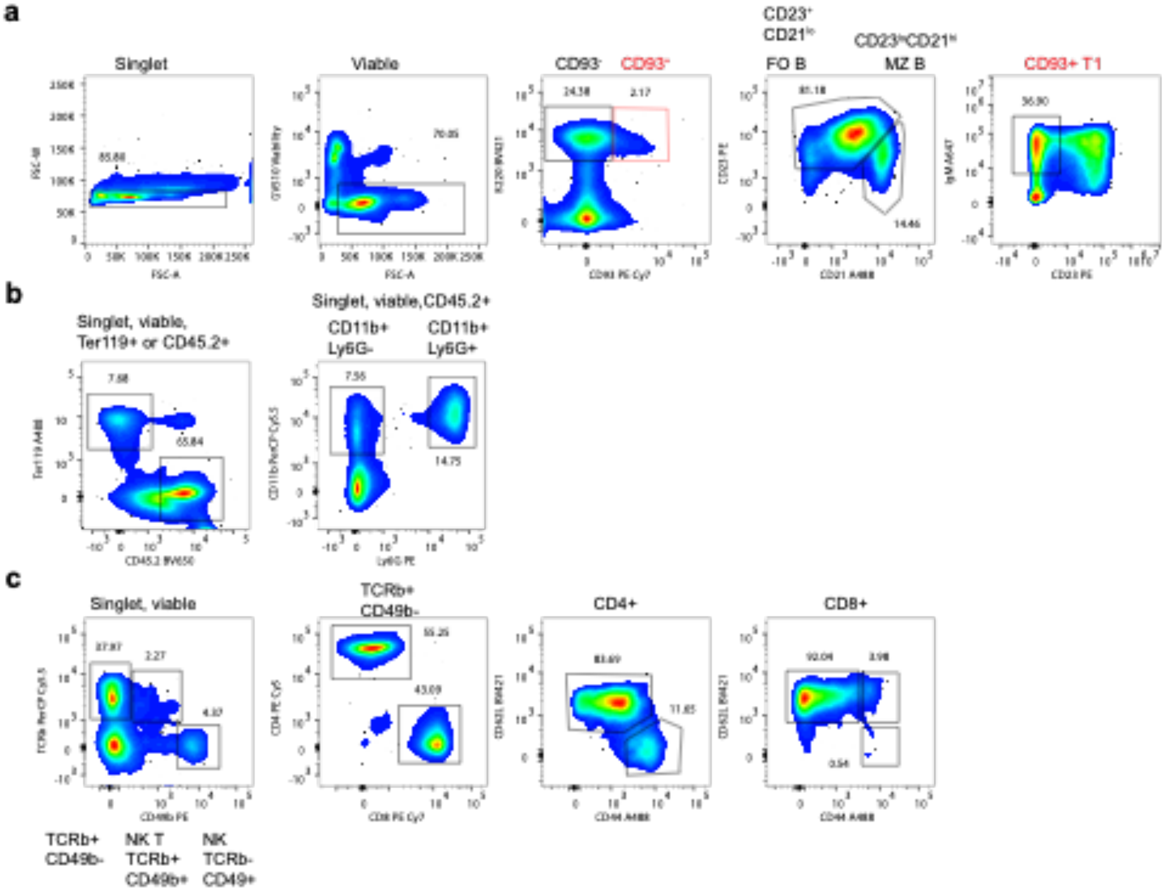

Supplementary Figure 15. Flow cytometry gating strategy.

a Depicted is gating strategy for B cell subsets, demonstrating gating for the removal of doublet events (Singlet), dead cells (Viable), separation of immature ($CD93^{+}$) and ($CD93^{-}$) B cells before identifying Marginal zone B cells ($CD93^{-}CD23^{lo}CD21^{hi}$), Follicular B cells ($CD93^{-}CD23^{+}CD21^{lo}$), or transitional 1 immature B cells ($CD93^{+}IgM^{+}CD23^{lo}$). **b** Gating strategy for erythroblasts ($CD45^{-}$ $Ter119^{+}$), leukocytes ($CD45^{+}$ $Ter119^{-}$) and neutrophils ($CD45^{+}$ $Ter119^{-}CD11b^{+}Ly6G^{+}$) after dead cell and doublet removal. **c** Gating strategy for NK, NK T cell and T cells as indicated. Naïve, effector, and memory T cell subsets were identified by CD62L and CD44 staining.

Supplementary Table 1: Antibodies used in study.

Antibody name	Clone	Supplier	Catalog number
CD16/32	2.4G2	Home made	NA
B220 BV421	RA3-6B2	BioLegend	103239
CD19 PE Cy7	ID3	BD Biosciences	552854
TCRb APC-Cy7	H57-597	BioLegend	109220
CD49b PE	HMa2	BD Biosciences	558759
CD62L eFluor 450	MEL-14	Thermo Fisher Scientific	48-3621-82
CD44 Alexa 488	IM7	Home made	NA
KLRG1 BV605	2F1/KLRG1	BioLegend	138419
CD4 PerCP Cy5.5	RM4-5	BD Biosciences	550954
CD8a Alexa 647	53-6.72	Home made	NA
Live Dead Ghost Viability 510	NA	Tonbo Biosciences	13-0870-T100
CD45.2 BV650	104	BioLegend	109836
Ter119	TER-119	BD Biosciences	557915
CD19 PerCP Cy5.5	ID3	Tonbo Biosciences	65-0193-U100
CD23 PE	B3B4	BD Biosciences	553139
CD21 Alexa 488	7E9	Home made	NA
CD93 PE Cy7	AA4.1	eBioscience	25-5892-82
B220 PE	RA3-6B2	BD Biosciences	553090
Ly6C APC Cy7	HK1.4	BioLegend	128026
CD4 PE Cy5	GK1.5	Tonbo Biosciences	55-0041-U100
CD8a PE Cy7	53-6.7	Tonbo Biosciences	60-0081-U100
CD62L PE	MEL-14	BioLegend	104407
CD44 Alexa 488	IM7	Home made	NA
TCRb PerCP Cy5.5	H57-597	BioLegend	109228
CD11b PerCP Cy5.5	M1/70	Tonbo Biosciences	65-0112-U100
Ly6G PE	IA8	BioLegend	127608
CD62L PE Cy5	MEL-14	BioLegend	104410
CD19 Pac Blue	6D5	BioLegend	115523
CD49b APC	HMa2	BD Biosciences	558295
CD68 Alexa 647	FA-11	BioLegend	137004
MHCII BV605	M5/114 15.2	BioLegend	107639
CD11c PE Cy7	N418	eBioscience	25-0114-82
TCRb APC	H57-597	Tonbo biosciences	20-5961-U100
IgD^b PE	217-170	BD Biosciences	553511
B220 PE Cy7	RA3-6B2	BD Biosciences	552772
B220 BUV737	RA3-6B2	BD Biosciences	612839
CD21 BV711	7E9	BioLegend	123435
Ter119 APC Fire 750	TER119	BioLegend	116250

**FlowCytomix Cytokine assay**		
IFN-γ bead	eBioscience	BMS822FF
IFN-γ biotin	eBioscience	BMS822FF
CXCL10 (IP-10) bead	eBioscience	BMS86018FF
CXCL10 (IP-10) biotin	eBioscience	BMS86018FF
TNF bead	eBioscience	BMS8607/2FF
TNF biotin	eBioscience	BMS8607/2FF
CXCL1 bead	eBioscience	BMS86019FF
CXCL1 biotin	eBioscience	BMS86019FF
IL-10 bead	eBioscience	BMS822FF
IL-10 biotin	eBioscience	BMS822FF
MCP3 bead	eBioscience	BMS821FF
MCP3 biotin	eBioscience	BMS821FF
**ELISA KIT**		
MCP3	Invitrogen	BMS6006INST
CXCL10	Invitrogen	BMS6018MST
IL-6	BD Biosciences	555240
IL-18	MBL	7625

Supplementary Table 2. PCR primers for genotyping

Gene	Forward	Reverse	Cut Enzyme
Tlr7 wt	agggtatgccgccaaatctaaag	acctttgtgtgctcctggac	N/A
Tlr7 KO	tcattctcagtattgttttgcc	acctttgtgtgctcctggac	N/A
Tlr9^{CpG11}	caccaatgcctttcagaacc	gccatctgagcgtgtacttg	N/A
Unc93b1 ^{3d}	gggagttaataccaggagacccag	atgctaacagttctgagccacctag	BstAp1
Tmem173 ^{gt}	cccaattctcttgtgctcacact	cgctgttggaataaacccgattcttg	BtsC1
Pld3	gcacatgcacacacacacaaag	tacaatgagggccaggtaagtg	Box1
Pld4^{thss}	aggatctctggaagagcagttg	cacatagcctacaaccctgtga	HpyCH4III
Tlr9 WT (BALB/c)	tccaacctgcggcagctgaac	cttcctcgtgctttacggtatcg	N/A
Tlr9 KO (BALB/c)	gttccctgtccatgaagagaacgcg	cttcctcgtgctttacggtatcg	N/A

Supplementary Table 3. Small liver RNA read length distributions. Two-month-old mice.

2 mo old	C57BL/6			*Tlr9*$^{CpG11/CpG11}$			*Tlr9*$^{CpG11/CpG11}$*Pld3*$^{–/–}$ *Pld4*$^{–/–}$			*Unc93b1*$^{3d/3d}$*Pld3*$^{–/–}$ *Pld4*$^{–/–}$		
length nt	B6-1	B6-2	B6-3	Tlr9KO-1	Tlr9KO-2	Tlr9KO-3	T9P3P4-1	T9P3P4-2	T9P3P4-3	UncP3P4-1	UncP3P4-2	UncP3P4-3
1	221	1285	1019	747	2445	1257	2041	306	1548	1497	1395	1897
2	163	757	709	607	1480	702	3720	2962	2581	1732	1662	1848
3	81	479	388	330	935	522	33257	20608	25157	9505	9917	10243
4	189	594	694	608	1215	616	105955	63099	91693	40819	45490	45171
5	437	1570	2607	2055	3564	1702	335797	151573	280356	155307	185761	185358
6	837	3607	5208	3677	8073	4429	353926	143743	300131	174405	222240	222837
7	1519	7085	9606	5439	14128	9343	124980	42021	117433	63462	81493	85820
8	2596	14193	17413	7546	26476	17641	99052	44064	100884	50005	59563	66059
9	2946	14597	17551	8654	28653	17843	75940	41031	77773	37890	43172	50530
10	4032	16110	19248	11298	32451	19479	100295	52657	104535	46299	54895	57915
11	5714	22989	25355	14634	45123	25392	112048	51719	128430	61084	69607	72172
12	8140	36930	38117	23210	72687	39831	119276	49193	146944	73809	78012	93372
13	7483	25398	27479	18326	52945	28069	115965	51066	159715	67878	70121	84272
14	11327	30105	33201	27248	65319	31938	99278	48143	158391	62336	63705	72883
15	14579	38438	42106	30042	73226	47557	91776	48165	158059	60515	61714	70573
16	43821	88051	81884	65414	164504	80342	160440	66508	219084	129674	119115	133183
17	40948	105925	98015	80814	200610	92308	143475	65101	215798	131775	126263	128725
18	62374	72167	83269	80393	144883	71822	114376	62103	209277	85082	97326	106843
19	108804	112805	158278	100658	255624	97543	161477	73470	237364	174021	164469	138390
20	150954	400492	341704	380193	812309	326087	399357	107301	441576	566672	494102	440932
21	112056	349503	298123	244674	634209	270354	196943	87179	249780	256213	239091	233422
22	205043	489473	506698	342288	912211	382829	327101	176164	396235	435525	370145	342043
23	117885	350748	306923	247768	619374	286138	265747	124922	330621	297651	294075	312002
24	57776	223008	201951	203689	416768	186836	137064	64489	214558	173819	152967	158085
25	37567	119154	104913	90787	202998	95912	127516	52378	197901	132232	129179	133898
26	19090	51939	51759	47402	97854	45123	72823	40003	143560	67718	68191	63657
27	19078	44924	50496	52668	108090	40856	61572	35013	130217	54877	62514	57938
28	11797	30332	32890	30945	58821	26628	50281	29500	118521	40589	46220	42131
29	15125	37647	43957	52050	81690	33609	55416	29254	117321	49891	58413	51689
30	17762	35927	43692	36073	66958	32330	51862	27711	123964	36621	43890	39252
31	40914	60521	76762	80938	124358	65163	99654	60575	198301	68169	81717	75483
32	113798	193682	282983	321025	402714	180040	360386	215823	721757	214568	246657	182847
33	23249	48619	63960	50254	87794	48505	92447	41265	177076	155831	173976	74216
34	18927	49679	55647	42870	86228	46682	58562	38799	151878	40080	48083	45538

Supplementary Table 4. Small liver RNA read length distributions. Two-week-old mice.

2 wk old	C57BL/6			*Pld3*$^{-/-}$			*Pld4*$^{-/-}$			*Pld3*$^{-/-}$*Pld4*$^{-/-}$		
length nt	1- B6	2- B6	3- B6	PLD3 KO-1	PLD3 KO-2	PLD3 KO-3	PLD4 KO-1	PLD4 KO-2	PLD4 KO-3	PLD3/4 DKO-1	PLD3/4 DKO-2	PLD3/4 DKO-3
1	1117	1073	693	1053	526	1166	637	2712	392	2653	1482	2581
2	1117	360	254	559	353	475	185	2589	630	1133	616	1074
3	284	245	161	404	230	435	125	2378	103	7170	2339	7825
4	300	264	151	1724	1125	2232	727	5087	112	28319	10272	26933
5	563	687	410	7876	4500	10717	3182	13221	327	87629	35177	82720
6	1559	1842	891	6956	3946	10636	6173	77780	800	105769	41840	106249
7	2698	2749	1534	5577	3639	7460	12182	68516	1180	62994	31427	80968
8	5305	4203	2902	6767	4984	8619	15579	55957	2390	49865	27498	69158
9	6884	4969	4011	7906	5968	9681	15606	75669	2692	40733	18000	46421
10	9940	6152	4942	7551	6179	9379	15424	93591	4092	44252	19867	47847
11	11206	6203	5329	7404	6528	10060	18894	98383	4612	54314	26374	61243
12	14464	9638	7981	12861	10722	16565	26243	117458	5977	71563	43886	85851
13	25419	9718	10080	11807	13498	16700	26927	129861	10950	68577	44231	91499
14	28527	15170	14906	15006	13463	18790	22212	111334	10988	67304	44114	88598
15	37297	17880	16924	17538	16974	22583	37227	91041	13276	88563	65958	107155
16	47912	31460	29083	24086	22348	33399	39262	110773	18120	71436	55629	98993
17	63310	36879	33630	31008	26403	40517	44197	112199	23150	83837	65889	117125
18	150650	60890	51043	57500	67995	74769	52957	145123	52336	136600	81115	137835
19	90193	60487	57789	63769	51288	55564	62622	131685	37036	115580	84418	151403
20	214390	132161	117652	92685	103271	179697	180511	290020	83569	216838	187585	294322
21	192502	91330	74157	59485	81012	93110	181573	188162	65694	156963	135515	214251
22	285461	161678	136531	124130	141054	161248	142967	319564	91029	243034	226203	367531
23	227567	143997	103545	98435	116554	172106	117716	302206	84366	191033	163123	264603
24	182535	105687	82897	94051	89108	124637	70275	175601	70382	146528	129227	200326
25	217933	54919	59943	43211	134730	72381	42533	124263	107746	87807	77328	110190
26	53817	32587	29522	28443	30019	37992	33825	72903	22783	58961	49256	79479
27	61204	44967	36832	41817	36176	44534	33407	90388	27649	68076	44016	68610
28	52704	30613	31709	30101	32634	39332	28288	64367	24165	51984	40952	57498
29	62577	37771	36320	35924	41316	39487	28955	66654	29356	54345	35726	52989
30	65898	33393	38785	26983	36147	37245	32763	68682	27925	56071	41831	55827
31	103695	56989	57472	43846	65470	73122	52244	104643	49921	75511	50840	68613
32	127631	173699	126497	81160	94052	124486	165060	261358	54851	145064	87625	144704
33	86697	59737	60916	40065	46703	57582	41375	111491	36063	48506	34887	47106
34	81425	53768	51879	43976	54239	66014	42265	128875	34023	54177	34587	46687
