# Supplementary material for: Cleavage of DNA and RNA by PLD3 and PLD4 limits autoinflammatory triggering by multiple sensors
Source: Nat Commun. 2021 Oct 7;12:5874. doi: 10.1038/s41467-021-26150-w (PMC8497607; doi:10.1038/s41467-021-26150-w)
Supplement: Supplementary file 2 — Description of Additional Supplementary Files [file 41467_2021_26150_MOESM2_ESM.pdf]

Description of Additional Supplementary Files

File Name: Supplementary Data 1
Description: Excel file with RNAseq data analysis for Supplementary Fig 14.
