# Supplementary material for: Cleavage of DNA and RNA by PLD3 and PLD4 limits autoinflammatory triggering by multiple sensors
Source: Nat Commun. 2021 Oct 7;12:5874. doi: 10.1038/s41467-021-26150-w (PMC8497607; doi:10.1038/s41467-021-26150-w)
Supplement: Supplementary file 4 — Reporting Summary [file 41467_2021_26150_MOESM4_ESM.pdf]

nature research

Corresponding author(s): David Nemazee

Last updated by author(s): 2021/07/28

Reporting Summary

Nature Research wishes to improve the reproducibility of the work that we publish. This form provides structure for consistency and transparency in reporting. For further information on Nature Research policies, see our Editorial Policies and the Editorial Policy Checklist.

Statistics

For all statistical analyses, confirm that the following items are present in the figure legend, table legend, main text, or Methods section.

n/a	Confirmed	
☐	☒	The exact sample size (*n*) for each experimental group/condition, given as a discrete number and unit of measurement
☐	☒	A statement on whether measurements were taken from distinct samples or whether the same sample was measured repeatedly
☐	☒	The statistical test(s) used AND whether they are one- or two-sided<br>*Only common tests should be described solely by name; describe more complex techniques in the Methods section.*
☒	☐	A description of all covariates tested
☐	☒	A description of any assumptions or corrections, such as tests of normality and adjustment for multiple comparisons
☐	☒	A full description of the statistical parameters including central tendency (e.g. means) or other basic estimates (e.g. regression coefficient) AND variation (e.g. standard deviation) or associated estimates of uncertainty (e.g. confidence intervals)
☐	☒	For null hypothesis testing, the test statistic (e.g. *F*, *t*, *r*) with confidence intervals, effect sizes, degrees of freedom and *P* value noted<br>*Give P values as exact values whenever suitable.*
☒	☐	For Bayesian analysis, information on the choice of priors and Markov chain Monte Carlo settings
☒	☐	For hierarchical and complex designs, identification of the appropriate level for tests and full reporting of outcomes
☒	☐	Estimates of effect sizes (e.g. Cohen's *d*, Pearson's *r*), indicating how they were calculated

Our web collection on statistics for biologists contains articles on many of the points above.

Software and code

Policy information about availability of computer code

Data collection	BD Diva 8.0.2, Softmax Pro 6.2.1, SpectroFlo 2.2.0.3 were used in flow cytometry data collection.
Data analysis	Analysis of differential gene expression was carried out using the program DEGSeq2 or Edge v. Flow cytometry data was analyzed with FlowJo 10.7.1, FCS Express 7.06.0015. Image data was analyzed using ImageJ 2.1.0/1.53C. Prism 8.4.3 was used for analyzing a range of data types.

For manuscripts utilizing custom algorithms or software that are central to the research but not yet described in published literature, software must be made available to editors and reviewers. We strongly encourage code deposition in a community repository (e.g. GitHub). See the Nature Research guidelines for submitting code & software for further information.

Data

Policy information about availability of data

All manuscripts must include a data availability statement. This statement should provide the following information, where applicable:

- Accession codes, unique identifiers, or web links for publicly available datasets
- A list of figures that have associated raw data
- A description of any restrictions on data availability

An Excel file showing raw data and statistical analyses for Figs. 1,2,4-8 and Supplementary Figs 1,4,5,6,7,8 is available upon request. Excel files with normalized gene counts are available by request for Figure 5 a,b and for Supplemental Figure 13.

Field-specific reporting

Please select the one below that is the best fit for your research. If you are not sure, read the appropriate sections before making your selection.

- [x] Life sciences
- [ ] Behavioural & social sciences
- [ ] Ecological, evolutionary & environmental sciences

For a reference copy of the document with all sections, see nature.com/documents/nr-reporting-summary-flat.pdf

Life sciences study design

All studies must disclose on these points even when the disclosure is negative.

Sample size

In determining the number of mice to be used in these experiments, we referred to "Statistical aspects of planning and design of immunological experiments", Elton, R.A. and McBride, W.H. In The Handbook of Experimental Immunology 4th edition, 1986, Vol 4, Chapter 131, pp131.1-131.6, Blackwell, Oxford. To provide a p<0.05 with 80% power in comparing two means with similar sample sizes and normal distributions, the formula used is:
n= 20(S.D.)^2 where n, S.D., and d are the sample size, standard deviation of
d^2
measurements and the differences in the experimental and control means. The referenced text notes that, for added stringency, 20 would be replaced by 30. Because in measurements of relevant parameters of leukocyte function the standard deviation approximates the mean, in order to detect a two-fold difference between experimental groups a sample size of 5-7 mice is needed per group. Usually, additional redundancy was added, such as independent experiments.

Effect sizes in flow cytometry as low as .05 can be measured with a probability of 80% with as few as 100 gated cells (FInak et al PMID:26861911). We typically stain at least a million cells and analyze between 50000 and 500000 cells / sample so we expect to be more than adequately powered for all but the most rare cell populations..

In vitro DC stimulation experiments were performed in 2-3 technical replicates, performed at least twice in separate experiments with similar results. Sample sizes were sufficient to detect differences between groups because these often exceeded a 10-fold difference with SD of less than 10%.

Data exclusions

No data were excluded from any analysis.

Replication

Fig 1: Survival of Tlr9-CpG11/CpG11Pld3-/-Pld4-/- n= 20, Pld3-/-Pld4-/- n=17, Unc93b13d/3dPld3-/-Pld4-/- n= 18, Unc93b1+/3dPld3-/-Pld4-/- n=16. Fig1c-k between four and six animals of each genotype analyzed, experiment performed at least twice. Fig 2: 3-5 mice per group were analyzed once. Fig 3 reactions performed at least twice. Fig 4 a,b experiments were performed at least twice. Fig 4 c-f: Experiment performed repeatedly at 8 different substrate concentrations (see Fig S10). Figure 5 a,b sequences derived from three liver samples each genotype. Figure 5c performed twice, lysosomes isolated from a different source tissue each time.
Fig 6: In a,b measurements were made in triplicate cultures. In c, duplicate cultures were analyzed. These results were repeated at least three times with similar results (also see Supplementary Fig. 11). Fig 7: b (3 mice/group); d (4-6 mice/group); FIg 8: 5-8 mice/genotype were tested.
All experiments presented were reliably reproduced at least twice except Figure 2, which was performed once, with multiple mice of each genotype.

Randomization

Both male and female animals of genetically modifed genotypes were used in all experiments. They were always age and sex matched with each other in each experiment. Wherever possible, experimental animals were compared to littermate controls.No formal randomization method was used.

Blinding

Genotypes were blinded in the liver histology pathology analyses shown. Genotypes were not blinded in other experiments because they were not deemed to be influenced by human interpretation.

Reporting for specific materials, systems and methods

We require information from authors about some types of materials, experimental systems and methods used in many studies. Here, indicate whether each material, system or method listed is relevant to your study. If you are not sure if a list item applies to your research, read the appropriate section before selecting a response.

Materials & experimental systems

n/a	Involved in the study	
☐	☒	Antibodies
☐	☒	Eukaryotic cell lines
☒	☐	Palaeontology and archaeology
☐	☒	Animals and other organisms
☒	☐	Human research participants
☒	☐	Clinical data
☒	☐	Dual use research of concern

Methods

n/a	Involved in the study	
☒	☐	ChIP-seq
☐	☒	Flow cytometry
☒	☐	MRI-based neuroimaging

Antibodies

Antibodies used

Antibodies used are described in Supplementary Table 1 as follows: (Antibody specificity/ name/ Clone Supplier/ Catalog number)
CD16/32 2.4G2 Home made NA
B220 BV421 RA3-6B2 BioLegend 103239
CD19 PE Cy7 ID3 BD Biosciences 552854
TCRb APC-Cy7 H57-597 BioLegend 109220
CD49b PE HMa2 BD Biosciences 558759
CD62L eFluor 450 MEL-14 Thermo Fisher Scientific 48-3621-82
CD44 Alexa 488 IM7 Home made NA
KLRG1 BV605 2F1/KLRG1 BioLegend 138419
CD4 PerCP Cy5.5 RM4-5 BD Biosciences 550954
CD8a Alexa 647 53-6.72 Home made NA
Live Dead Ghost Viability 510 NA Tonbo Biosciences 13-0870-T100
CD45.2 BV650 104 BioLegend 109836
Ter119 TER-119 BD Biosciences 557915
CD19 PerCP Cy5.5 ID3 Tonbo Biosciences 65-0193-U100
CD23 PE B3B4 BD Biosciences 553139
CD21 Alexa 488 7E9 Home made NA
CD93 PE Cy7 AA4.1 eBioscience 25-5892-82
B220 PE RA3-6B2 BD Biosciences 553090
Ly6C APC Cy7 HK1.4 BioLegend 128026
CD4 PE Cy5 GK1.5 Tonbo Biosciences 55-0041-U100
CD8a PE Cy7 53-6.7 Tonbo Biosciences 60-0081-U100
CD62L PE MEL-14 BioLegend 104407
CD44 Alexa 488 IM7 Home made NA
TCRb PerCP Cy5.5 H57-597 BioLegend 109228
CD11b PerCP Cy5.5 M1/70 Tonbo Biosciences 65-0112-U100
Ly6G PE IA8 BioLegend 127608
CD62L PE Cy5 MEL-14 BioLegend 104410
CD19 Pac Blue 6D5 BioLegend 115523
CD49b APC HMa2 BD Biosciences 558295
CD68 Alexa 647 FA-11 BioLegend 137004
MHCII BV605 M5/114 15.2 BioLegend 107639
CD11c PE Cy7 N418 eBioscience 25-0114-82
TCRb APC H57-597 Tonbo biosciences 20-5961-U100
IgDb PE 217-170 BD Biosciences 553511
B220 PE Cy7 RA3-6B2 BD Biosciences 552772
B220 BUV737 RA3-6B2 BD Biosciences 612839
CD21 BV711 7E9 BioLegend 123435
Ter119 APC Fire 750 TER119 BioLegend 116250.

Antibodies were validated and used at 1/200, in a 100uL volume, 4x106 cells.

Validation

All antibodies used have been tested and validated for the species and application (Flow-cytometry and/or immuno-fluorescence) as stated on the website of the manufacturers. https://www.biolegend.com/, https://www.thermofisher.com/, https://www.bdbiosciences.com/, https://tonbobio.com. Antibodies that were conjugated with fluorophores in our laboratory were validated by titration in flow cytometry analyses, using wild type mouse tissue where the expected frequencies were well known.

Eukaryotic cell lines

Policy information about cell lines

Cell line source(s)

The eukaryotic cell line HEK293S GnTI was used to generate recombinant soluble PLD3 and PLD4 in this study. This cell line

	was obtained from the American Type Culture Collection. HEK293 cells expressing human TLR9 or mouse TLR13 and an NFkB reporter system were initially purchased from Invivogen (cat # hkb-htlr9, hkb-mtlr13). The PLD3 gene was ablated using Crispr/Cas9 as previously reported and verified using Sanger sequencing.
Authentication	HEK293S GnTI was authenticated by the American Type Culture Collection to lack mycoplasma infection. HEK293 cells expressing human TLR9 or mouse TLR13 and an NFkB reporter system were authenticated by us for PLD3 expression and NFkB reporter activity.
Mycoplasma contamination	The 2HEK293S GnTI cell line has not been tested for mycoplasma by our laboratory. HEK293 cells expressing human TLR9 or mouse TLR13 and an NFkB reporter system were not tested for mycoplasma.
Commonly misidentified lines (See ICLAC register)	The 2HEK293S GnTI cell line cell line was used to produce recombinant proteins that were later purified, so the genetic origin of the cell line should have little influence on the characteristics of the purified protein used.

Animals and other organisms

Policy information about studies involving animals; ARRIVE guidelines recommended for reporting animal research

Laboratory animals	The following laboratory animal strains were used. Pld4-/- animals were generated at The Scripps Research Institute in a 129/Ev strain ES cell and backcrossed to C57BL/6J. Pld3-/- mice were generated at The Scripps Research Institute on the C57BL/6J background. Tlr9 deficient and Unc93b3d/3d mice were generated by ENU mutagenesis on the C57BL/6 background. Tlr7-/- mice were strain B6.129S1-Tlr7tm1Flv/J from Jackson Labs. Ifng-/- mice were strain B6.129S7-Ifngtm1Ts/J from Jackson labs. Tlr9-CpG11/CpG11 mice were C57BL/6J-Tlr9M7Btlr/Mmjax (CpG11) from the Jackson Laboratory. Tlr9–/– mice on the BALB/c background were donated by Ann Rothstein. Ifnar1-/- mice were strain B6.129S2-Ifnar1tm1Agt/Mmjax from Jackson labs. Unc93b3d/3d mice were obtained from Dr Bruce Beutler. Both male and female mice of ages ranging from 2 months to 8 months were used. Pld3-/-Pld4-/- mice or their littermates were generated by interbreeding the Pld3-/- and Pld4-/- lines and were studied between 16 and 21 days of age. Mice were housed in specific pathogen free conditions at 68-72°F and 30-70% humidity, with 6pm-6am nocturnal dark/light cycle.
Wild animals	No wild animals were used in this study.
Field-collected samples	No field samples were used in this study.
Ethics oversight	Study protocols were subject to approval by the Institutional Animal Care and Use Committee of The Scripps Research Institute. Protocol number 09-0105-4/5.

Note that full information on the approval of the study protocol must also be provided in the manuscript.

Flow Cytometry

Plots

Confirm that:

- [x] The axis labels state the marker and fluorochrome used (e.g. CD4-FITC).
- [x] The axis scales are clearly visible. Include numbers along axes only for bottom left plot of group (a 'group' is an analysis of identical markers).
- [x] All plots are contour plots with outliers or pseudocolor plots.
- [x] A numerical value for number of cells or percentage (with statistics) is provided.

Methodology

Sample preparation	Tissue suspensions were generated by mashing between frosted glass slides. Bone marrow was released from tibia and femurs by a mortar and pestle crushing method. Red blood cells were lysed with ammonium chloride (0.83%) and cells filtered to generate single cell suspensions. Isolated cells were washed with FACS Buffer (1x PBS with 1% BSA, 10 mM HEPES pH 8, 1 mM EDTA) prior to incubation with 1 ug 2.4G2 antibody (Fc block) on ice for 15 minutes before staining.
Instrument	Data collection was performed using BD LSRII and Cytek Auroa analyzers.
Software	Flow cytometry data was acquired on BD LSRII machines using BD Diva software. Data analysis was performed using Flow Jo (Treestar) software or FCS Express 6.
Cell population abundance	Cell population abundance was determined by flow cytometry electronic gating of samples and multiplying population proportion by total tissue cell count (as determined by Z2 Coulter particle counter) after dispersion and erythrocyte lysis.
Gating strategy	Samples were gated to exclude doublets based on FSC-A vs FSC-W and dead cells excluded by Ghost viability stain GV510. Live T cells were further characterized by CD4 or CD8 staining, and CD44 and CD62L staining. Marginal zone B cells were

gated on B220+CD93-CD23loCD21hi. Lymphocyte gating strategies shown in respective figures. Neutrophils and myeloid cells were gated on Ly6G+CD11b+ or Ly6G-CD11b+ respectively and these gates are outlined in Supplementary data 14.

☒ Tick this box to confirm that a figure exemplifying the gating strategy is provided in the Supplementary Information.
